# The mucosal-neural axis in chemotherapy-induced gastrointestinal toxicity

**DOI:** 10.3389/or.2026.1814601

**Published:** 2026-05-11

**Authors:** Mikaela R. Nemani, Sandra Rieger

**Affiliations:** 1 Department of Biology, University of Miami, Coral Gables, FL, United States; 2 Sylvester Comprehensive Cancer Center, Miller School of Medicine, University of Miami, Miami, FL, United States

**Keywords:** chemotherapy, CINV, clinical guidelines, DRG, MMP, mucositis, ROS

## Abstract

Chemotherapy-induced gastrointestinal toxicity (CIGT) is a common, dose-limiting complication of cancer therapy that manifests as mucositis, nausea, vomiting, diarrhea, abdominal pain, and weight loss. Epithelial injury, barrier disruption, oxidative stress, and inflammatory signaling are well-established features of CIGT affecting the mucosa, however, these mechanisms do not fully explain the persistence of neurosensory symptoms that can outlast histologic recovery. In particular, the pathways linking epithelial damage to sustained activation of enteric and extrinsic sensory circuits remain incompletely defined. Here, we propose that CIGT involves coordinated interactions between the injured intestinal epithelium and peripheral neural pathways, in which epithelial-derived signals and inflammatory mediators drive neuronal hyperexcitability and sustained sensory dysfunction. Serotonergic signaling from enterochromaffin cells represents a well-established mechanism linking epithelial activity to vagal afferents and nausea. In contrast, the contributions of inflammatory mediators, oxidative stress, barrier dysfunction, and microbial dysbiosis to enteric and dorsal root ganglion (DRG) neuron sensitization remains less clearly defined. Current clinical management is largely palliative and targets downstream symptoms rather than upstream epithelial-to-neural interactions. A better understanding of how epithelial injury engages neural pathways may enable mechanism-based therapies that improve symptom control, preserve treatment intensity, and enhance patient outcomes.

## 1 Introduction

Chemotherapy-induced gastrointestinal toxicity (CIGT) originates from epithelial damage caused by cytotoxic effects on rapidly dividing cells. However, the clinical manifestations of CIGT, including nausea, vomiting, abdominal pain, and diarrhea, likely reflect not only epithelial injury but also alterations in epithelial-to-neural signaling that regulate gastrointestinal function. This is supported by the efficacy of therapies targeting neuronal signaling in acute emesis, such as 5-HT3 receptor antagonists used to treat chemotherapy-induced nausea and vomiting (CINV). Despite this, delayed symptoms and non-emetic manifestations, including abdominal pain and diarrhea, remain incompletely managed, and these treatments do not address the underlying epithelial injury. Together, this suggests that epithelial-to-neural crosstalk may contribute to disease pathophysiology. However, the mechanisms linking epithelial damage to sustained activation of enteric and extrinsic sensory pathways, including DRG neurons involved in pain and hypersensitivity, remain incompletely defined. We also consider the possibility that sensitized neurons further amplify CIGT and discuss therapeutic strategies targeting epithelial mechanisms.

### 1.1 Clinical features of chemotherapy-induced gastrointestinal toxicity

**FIGURE 1:**
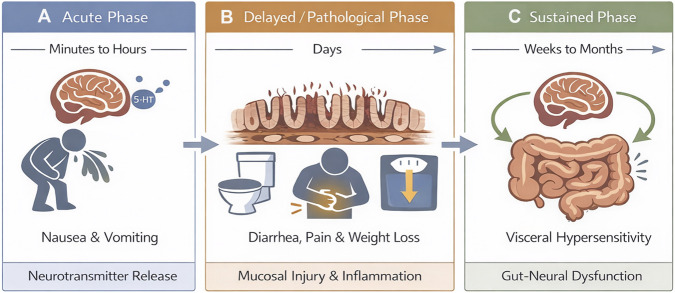
The temporal resolution of CIGT. The clinical presentation of CIGT follows a distinct temporal scale that reflects different underlying pathobiological processes. **(A)** Acute Phase: Immediate nausea and vomiting typically occur within minutes to several hours post-administration, driven primarily by rapid neurotransmitter release (i.e., serotonin). **(B)** Delayed/Pathological Phase: Within the next few days, cytotoxic injury to the intestinal crypts results in peak mucosal barrier breakdown and inflammation. This phase manifests clinically as diarrhea, abdominal pain, and significant weight loss. **(C)** Sustained Phase: Neurosensory dysfunction and visceral hypersensitivity often persist beyond histological epithelial recovery, suggesting a long-term sensitization of the gut-neural axis.

Chemotherapy-induced gastrointestinal toxicity (CIGT) is a comprehensive term encompassing a spectrum of adverse effects occurring as a direct or indirect consequence of cytotoxic chemotherapy. It is a complex pathology where symptoms manifest along a distinct temporal scale. While acute nausea and vomiting often occur rapidly following drug administration due to immediate neurotransmitter release, other features emerge as the underlying pathobiology evolves. The most prominent pathological hallmark is gastrointestinal mucositis, characterized by inflammation and ulcerative lesions of the mucosal lining (1, 2) (Figure 1). Following this initial phase, progressive mucosal damage gives rise to a constellation of debilitating clinical manifestations, including diarrhea, anorexia, and constipation, often accompanied by abdominal pain and significant weight loss (3–5). While these symptoms were traditionally attributed solely to epithelial injury. The pathogenesis of CIGT is however now understood to involve a synergistic interplay of gut microbiota dysbiosis (6, 7), dysregulated inflammatory responses (1, 6), and oxidative stress (7). Notably, the unregulated release of serotonin from damaged enterochromaffin cells acts on enteric and extrinsic sensory circuits, driving the neural dysfunction that characterizes CIGT (8). While the role of the dorsal root ganglion (DRG), which also innervate the gut wall, is well-established in somatic neuropathy affecting the extremities, their specific contributions to abdominal pain and nausea in chemotherapy patients remains an important area for further exploration.

#### 1.1.1 Prevalence and dose-dependency of CIGT

The prevalence of CIGT among patients undergoing chemotherapy is remarkably variable, affecting between 5% and 100% of individuals (2, 9–12). This wide range is not arbitrary but is directly dependent on several key factors, most notably the specific chemotherapeutic agent used, the administered dose, and the treatment regimen (9, 13–15) (Table 1). An estimated death rate of 7.5% has been reported as a result of non-selective toxicities that occur in chemotherapy patients (16), underscoring the severe impact these agents have on cancer treatment outcomes. Thus, better management of these side effects or the prevention of toxicities altogether will be essential to ensure therapeutic success.

**TABLE 1:** Incidence of commonly used chemotherapy agents and related CIGT symptoms.

Drugs/Combination regimen	Symptoms	Incidence per Study	Antiemetic drugs and treatment response rate	References (DOI)
Carboplatin + paclitaxel	Delayed CINV	36.30%	Triple prophylaxis with palonosetron or other 5-HT, receptor antagonist + dexamethasone + NK_1_ receptor antagonist (RA) (aprepitant): Low-dose olanzapine; trial showed 86.9% CR vs. 80.6% with placebo for acute CINV and ∼89% patients free of nausea, two-drug prophylaxis (5-HT, RA + dexamethasone) achieved ∼67% overall CR in an observational study	10.1186/s12885-021-07802-y
Carboplatin + pemetrexed	Delayed CINV	51.10%	Two-drug prophylaxis (5-HT, RA + dexamethasone) gave only ∼54% overall; triple prophylaxis with palonosetron + dexamethasone + aprepitant or olanzapine is recommended for improved delayed control	10.1186/s12885-021-07802-y
Paclitaxel	Nausea and vomiting	52%	Standard prophylaxis uses a 5-HT, receptor antagonist plus dexamethasone; in a prospective study of moderately emetogenic regimens the overall complete response rate was 64%, addition of an NK_1_ receptor antagonist or olanzapine improves control and is recommended for high-risk patients	NDA 020262/s-048
Paclitaxel	Diarrhea	38%	Diarrhea is managed with anti-diarrheal agents (e.g., loperamide) and hydration; antiemetic prophylaxis is not applicable	NDA 020262/s-048
Paclitaxel	Mucositis	31%	Mucositis management involves oral care, saline mouth rinses and mucosal protectants; antiemetic prophylaxis is not applicable	NDA 020262/s-048
Docetaxel + epirubicin	Nausea	39%	For anthracycline-taxane regimens, triple prophylaxis with NEPA (netupitant + palonosetron) plus dexamethasone achieved 85.7% complete response vs. 64.4% with aprepitant-based therapy, later cycles achieved >90% CR.	10.1023/a:1011663821703
Docetaxel + epirubicin	Mucositis	36%	Supportive care (good oral hygiene, topical analgesics); specific anti emetic therapy is not applicable	10.1023/a:1011663821703
Irinotecan	Vomiting (acute and delayed onset)	7% and 9%	Prophylaxis with a 5-HT, RA + dexamethasone produced 86% complete response in the first 24 h and 82% delayed-phase CR; overall CR was 77%	10.1007/s00520-011-1286–6
Irinotecan	Delayed nausea	34%	The same study reported a 34% incidence of delayed nausea; two-drug prophylaxis (5-HT, RA + dexamethasone) produced 82% delayed-phase complete response	10.1007/s00520-011-1286–6
Platinum-based chemotherapy (combined incidence)	Nausea	52%	For highly emetogenic cisplatin or moderately emetogenic carboplatin, triple therapy with 5-HT, receptor antagonist, dexamethasone and NK_1_ RA (tolanzapine) is standard. Meta-analyses show >80% acute complete response and about 70% overall CR.	10.3390/curroncol32060325
Platinum-based chemotherapy (combined incidence)	Vomiting	37%	Adding an NK_1_ receptor antagonist to 5-HT, RA + dexamethasone improved no-vomiting rates from 76.98% to 91.3% in cisplatin regimens; complete response rates were >80% in the acute phase	10.3390/curroncol32060325
Cisplatin	Acute versus delayed vomiting	>90% vs. 45%	For cisplatin (>70 mg/m^2^) triple prophylaxis with 5-HT, RA + dexamethasone and NK_1_ RA:olanzapine yielded >80% acute CR and ∼70% overall CR; addition of NK_1_ RA improved no-vomiting rates to 91.3%	10.3390/curroncol32060325
Carboplatin	Vomiting	19%	Carboplatin is considered moderately emetogenic. Two-drug prophylaxis (5-HT, RA + dexamethasone) achieved ∼64–67%; addition of NK_1_ RA or olanzepine improved CR to 80%	10.3390/curroncol32060325
Oxaliplatin	Vomiting	21%	For oxaliplatin-based regimens, two-drug prophylaxis (5-HT, RA + dexamethasone) provided 90% acute-phase CR but delayed-phase CR dropped to 54% when a third drug was not given. Addition of NK_1_ RA improves delayed control	10.3390/curroncol32060325
Vincristine	Emesis	<10%	Vincristine is minimally emetogenic; routine prophylaxis is usually not required. Single-dose 5-HT, RA + dexamethasone achieves >90% control of acute emesis (guideline-based estimate)	10.1200/JCO.2017.74.4789
Anthracycline + cyclophosphamide	Emesis	54.50%	In breast-cancer patients receiving doxorubicin/cyclophosphamide, triple prophylaxis with NEPA + dexamethasone achieved 85.7% complete response vs. 64.4% with aprepitant-based therapy; subsequent cycles showed CR >90%	10.1200/JCO.2017.74.4789

Abbreviations: CINV, chemotherapy-induced nausea and vomiting; RA, receptor antagonist; CR, complete response; 5-HT, serotonin.

Certain classes of chemotherapeutic agents are particularly notorious for inducing severe gastrointestinal toxicities, such as Grade 3 and Grade 4 diarrhea. Although across clinical trials and regulatory reports, any-grade diarrhea is common, grade 3 or four diarrhea occurs in roughly 20%–36% depending on dose and schedule (17, 18). One such agent, causing severe diarrhea is irinotecan (CPT-11, a topoisomerase-I inhibitor), which is strongly associated with delayed diarrhea (19, 20). For instance, CPT-11 induced diarrhea occurs early in about 51% of patients, whereas late occurrences affect 88%. And 31% of the patients display severe diarrhea (21). Fluoropyrimidines are another class frequently implicated in chemotherapy-related diarrhea. For instance, randomized clinical trials comparing bolus 5-FU/LV with capecitabine reported that both arms showed clinically meaningful rates of diarrhea (22).

Other chemotherapeutic agents, such as cisplatin instead cause other GI distress symptoms. For instance, cisplatin is emetogenic, causing nausea and/or vomiting. In placebo-controlled or minimal-prophylaxis randomized trials, a majority of patients experienced these symptoms with cisplatin when antiemetics were omitted, underscoring its distinct emetogenic profile. This contrasts with diarrhea-inducing agents such as irinotecan or fluoropyrimidines, which primarily drive gastrointestinal mucosal injury rather than emetic pathway activation (23, 24).

#### 1.1.2 Impact of CIGT on patient survival

The consequences of CIGT are severe and multifaceted, impacting patient wellbeing, the feasibility of treatment, and ultimately, survival outcomes. The persistent and distressing symptoms of pain, nausea, vomiting, and diarrhea lead to a cascade of secondary complications, including malnutrition, anorexia, and severe weight loss. More than 63% of cancer patients undergoing chemotherapy experience unintentional weight loss, a condition directly linked to the symptoms of CIGT (1, 4). This results in poor nutritional status and is associated with poorer survival outcomes (25). The physical and emotional burden of these symptoms can lead to functional impairment, social isolation, and psychological distress, further eroding quality of life (QoL) (26).

Perhaps the most critical clinical consequence of CIGT is its role as a primary dose-limiting toxicity (2, 27). The severity of symptoms frequently forces clinicians to make the difficult choice of interrupting or altering the planned chemotherapy regimen (28). This manifests as treatment delays or dose reductions, which are common strategies to manage acute toxicity (29). This practice, while necessary for patient safety in the short term, has grave implications for treatment efficacy and survival outcomes. The concept of Relative Dose Intensity (RDI), the ratio of the delivered dose to the planned dose over a specific period, is a critical metric in oncology. A substantial body of clinical evidence demonstrates a direct, dose-dependent relationship between RDI and treatment outcomes, including progression-free and overall survival (30). Maintaining a relative dose intensity (RDI) of ≥80–85% is widely regarded as clinically meaningful, since lower RDI has been associated with inferior survival outcomes (31, 32). However, delays and dose reductions remain common in practice: for example, in first-line platinum-based chemotherapy for advanced NSCLC, ∼32% of patients had ≥7-day delays, ∼50% had ≥15% dose reductions, and ∼40% had RDIs of <85% (33). Treatment-limiting toxicities (including gastrointestinal and other toxicities) often contribute to these modifications, thereby reducing the likelihood of achieving the target RDI.

This underscores a central paradox of chemotherapy: while intended to eradicate malignant cells, its toxic effects on healthy tissues can necessitate treatment modifications that ultimately limit the ability to deliver therapy at its full intended intensity. CIGT is therefore not a peripheral “side effect” but a direct antagonist to the primary therapeutic goal. A clear sequence of events emerges where the administration of chemotherapy leads to CIGT, which in turn necessitates dose modifications. These modifications result in a compromised RDI, which is directly linked to reduced treatment efficacy and poorer survival rates (34). This reframes the management of CIGT from a matter of palliative care to a critical component of effective cancer treatment (35). An intervention that could successfully mitigate CIGT would not only improve patient QoL but could also enhance cancer survival rates, simply by enabling the delivery of the full, planned therapeutic dose of chemotherapy.

In its most severe forms, CIGT can be life-threatening, such as in patients with acute myeloid leukemia (16, 36). Chemotherapy in these patients can lead to neutropenic enterocolitis, a bacterial infection that causes profound damage to the mucosal barrier leading to necrotizing perforations, uncontrolled bleeding, abdominal abscess. Breakdown of the epithelial barrier allows for the translocation of luminal bacteria and their toxins into the bloodstream, which can lead to systemic infections and sepsis (37). Furthermore, the treatment delays necessitated by CIGT are independently associated with an increased risk of death. A meta-analysis across several major cancer types found that for every 4-week delay in initiating adjuvant or neoadjuvant treatment, the risk of mortality increases significantly (38). Thus, CIGT can contribute to fatal outcomes both directly, through complications like sepsis, and indirectly, by compromising the effectiveness of the cancer treatment itself.

### 1.2 Chemotherapeutic agents induce intestinal epithelial damage through shared cytotoxic mechanisms

**FIGURE 2:**
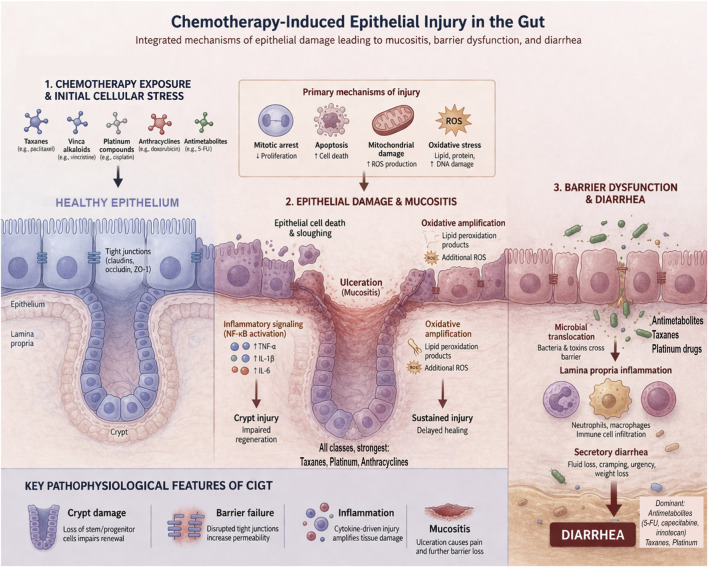
Chemotherapy-induced epithelial injury and barrier dysfunction in CIGT. This schematic illustrates the primary epithelial mechanisms known to underlie chemotherapy-induced gastrointestinal toxicity. Chemotherapeutic agents preferentially affect highly proliferative crypt cells but also induce damage in differentiated epithelial cells. Oxidative stress caused by ROS and mitochondrial dysfunction are main drivers. This leads to inflammatory signaling (e.g., TNF-α, IL-1β, IL-6) and oxidative amplification, causing epithelial injury and mucositis. Concurrently, tight junction proteins (e.g., claudins, occludin, ZO-1) are downregulated, resulting in increased epithelial permeability and barrier disruption. This facilitates microbial translocation and further inflammatory activation. The combined effects of epithelial injury, barrier dysfunction, and inflammation impair fluid absorption and promote secretion, ultimately leading to diarrhea and other gastrointestinal symptoms characteristic of CIGT.

Chemotherapeutic agents induce gastrointestinal toxicity through distinct primary mechanisms. However, despite these differences, they converge on a limited set of cellular stress pathways that compromise epithelial integrity and mucosal barrier function. The initial insult arises from direct interactions between chemotherapeutic agents and the intestinal epithelium, a tissue characterized by rapid and continuous self-renewal. These drugs are designed to target proliferating cancer cells but lack tissue specificity, rendering the rapidly renewing gut epithelium an inevitable site of collateral damage (35). The gastrointestinal epithelium undergoes complete renewal every 4–5 days, driven by highly proliferative stem and progenitor cells located at the base of intestinal crypts (39), making this compartment particularly susceptible to cytotoxic injury. Although different classes of chemotherapeutic agents have distinct molecular targets, including DNA, DNA-associated enzymes, and microtubules, they functionally converge on disruption of the cell cycle, leading to impaired proliferation and cell death within intestinal crypts (40, 41) (Table 2). In parallel, many of these agents induce reactive oxygen species (ROS) and mitochondrial dysfunction, which amplify epithelial damage by promoting apoptosis, inflammatory signaling, and loss of barrier integrity (Figure 2) (42). Here we provide an overview of major chemotherapeutic classes and their contributions to mucosal damage, highlighting shared effects on epithelial proliferation, apoptosis, and oxidative stress.

**TABLE 2:** Chemotherapy effects on intestinal mucosa.

Drug class	Example agents	Consequence in intestinal epithelial cells	References (DOI)
Taxanes	Paclitaxel, docetaxel	Mucositis, rapid crypt apoptosis followed by villus atrophy	10.1097/PAS.0b013e3181582331; 10.4143/crt.2009.41.4.196; 10.3760/cma.j.issn.0366-6999.2011.12.019
Platinum drugs	Cisplatin, carboplatin	CS: Intestinal apoptosis in both crypt and villus epithelium, ROS induced damage; CB: Intestinal mucositis resulting in weight loss, diarrhea, and infiltration of inflammatory cells	10.1016/j.nut.2011.09.007; 10.1038/s41419-019-1963–9
Vinca alkaloids	Vincristine, vinblastine	VC: Severe villous atrophy and occasional mucosal erosions; VB: Increase in lipoperoxidation and ROS production, significant depletion of enzymatic and non-enzymatic antioxidants, disruption of intracellular iron and calcium levels	10.1007/978-3-642–69928-3_15; 10.1016/j.toxrep.2017.04.006
Anthracycline	Doxorubicin, idarubicin	Diarrhea. Molecular hallmarks: Rapid decrease in proliferation and increase in apoptosis leading to villus height reduction, decreased mucosal permeability followed by recovery	10.1007/s00210-022-02311–6; 10.1111/bcpt.13851
Antimetabolites	Cytarabine (Ara-C)	Loss of mucosal integrity, necrosis and decreased villus length, reduced mucosal proliferation	10.1177/030089160909500114
Antimetabolites (pyrimidine analog)	5-Fluorouracil (5-FU)	Shortened villus height, disruption of crypts, loss of goblet cells due to early crypt apoptosis via caspase 3/8. TNF-α, IL-1β, and Nox1-dependent ROS formation	10.1152/ajpgi.00535.2011; 10.1590/acb370204
Topoisomerase I inhibitors	Irinotecan	Severe diarrhea and abdominal cramps. Molecular hallmarks: decreased proliferation in jejunum and colon, preceded by early apoptosis. Increased collagen deposits, collagen IV expression decrease in crypts, fibronectin expression decrease in jejunum, and altered epithelial cell kinetics correlated with changes in ECM	​
Alkylating agents	Cyclophos-phamide, ifosfamide	Necrosis, crypt progenitor depletion, intestinal protein loss	10.1038/bjc.1984.5; 10.1007/978-3-642–69928-3_15

Abbreviations: CS, cisplatin; CB, carboplatin.

#### 1.2.1 Microtubule-targeting agents

Microtubule-targeting agents, such as taxanes, have been well characterized with respect to their effects on cancer cells. They disrupt microtubule dynamics by stabilizing polymerized tubulin. Their cytotoxic effects are primarily attributed to interference with aberrant spindle formation and chromosome segregation defects (43–45). This triggers stress-response pathways leading to mitotic catastrophe and apoptosis (46–50). In the intestinal epithelium, which undergoes rapid and continuous renewal, these microtubule-modifying effects are expected to impact proliferation and self-renewal of epithelial cells within the crypt compartment. Consistently, histopathological analyses of gastrointestinal biopsies from patients treated with taxanes revealed characteristic microscopic features, including an accumulation of crypt cells in mitotic arrest and the formation of abnormal “ring mitoses”, providing direct evidence of drug effects on the epithelial cells (51).

The most prominent member of the taxane family, paclitaxel, induces mitotic inhibition and apoptosis in the intestinal epithelium (52, 53). Intravenous administration of paclitaxel in mice was shown to rapidly block mitotic divisions within 2–4 h, followed by apoptosis peaking at 6–8 h. Nevertheless, surviving crypt cells resumed proliferation after 24 h, indicating that crypt cells rapidly regenerate (52). Symptoms in patients manifest as nausea, vomiting, diarrhea, stomatitis (inflammation of the oral mucosa leading to mouth sores, pain, and eating difficulties) (54). Consistent with the fast regenerative capacity, paclitaxel causes mostly mild to moderate toxicity (grade 1-2), whereas docetaxel was found to be slightly more toxic, evoking grade 3 toxicity. Both paclitaxel and the semi-synthetic taxane, docetaxel (Taxotere), have been associated with diarrhea. It is estimated that ∼20–40% of patients treated with docetaxel suffer from diarrhea, with 5%–6% displaying severe symptoms (55). While diarrhea in paclitaxel treated patients is less common, it has been suggested that paclitaxel-based chemotherapy may predispose to severe *Clostridium difficile*-associated diarrhea (CDAD), particularly with prior antibiotic exposure and/or neutropenia (56). Microtubule disruption alone may not fully explain the broad epithelial phenotypes. *In vitro* studies using human intestinal epithelial cells demonstrated that besides cytoskeletal disruption, paclitaxel also induces the formation of ROS and mitochondrial damage together with downregulation of RhoGTPase (54). Thus, additional factors such as oxidative stress and inflammation are likely to play a role. Like taxanes, vinca alkaloids (vincristine, vinblastine) are anti-mitotic agents that target microtubules, however, these inhibit polymerization (57). This destabilizing action prevents the formation of a functional mitotic spindle and metaphase arrest (58). The cytocidal effect in the intestinal crypts was shown to be a cumulative result of interphase cell apoptosis and metaphase arrest of dividing cells (59). Taken together, microtubule-targeting agents converge on the disruption of crypt cell division as a central mechanism of gastrointestinal toxicity, but ROS and mitochondrial damage also play a role, which parallels its effects on cancer cells (60, 61).

#### 1.2.2 Platinum drugs

Opposed to microtubule-modifying drugs, platinum drugs (cisplatin, carboplatin, oxaliplatin) function as powerful alkylating-like agents in the treatment of a wide variety of cancers, including neuroblastoma, bladder, head and neck, lung, cervical, ovarian, testis, advanced gastrointestinal cancers, and rectal cancers (62). Upon entering the cell and undergoing hydrolysis, they become highly reactive and form covalent bonds with the N7 position of purine bases (adenine and guanine) in the DNA (63). This results in the formation of DNA adducts, primarily intra-strand and, to a lesser extent, inter-strand crosslinks. These crosslinks create significant structural distortions in the DNA (64), which physically obstruct the processes of DNA replication and transcription. This platinum-induced DNA damage activates DNA-damage surveillance pathways. In cells with functional p53, this response includes p53-mediated cell-cycle arrest to allow repair (65, 66). However, many cancers harbor TP53 mutations; in these cases, an inability to mount an effective G1/S checkpoint causes cells to progress with unrepaired crosslinks, ultimately leading to replication collapse and mitotic catastrophe and cell death (67, 68). Given the high rate of DNA replication, the progenitor cells of the intestinal crypts are exquisitely sensitive to this mechanism of action. For instance, studies in rat models show that a single dose of cisplatin (5 mg/kg) increased apoptosis in the intestinal crypts by 4-fold within 24–48 h. This damage preceded “crypt hypoplasia” (thinning), leading to the breakdown of the mucosal barrier (69). Common symptoms include abdominal pain, nausea, and vomiting, mucositis, delayed gastric motility, anorexia, and weight loss, besides electrolyte imbalances due to mucosal barrier loss (70). Often, these symptoms are persistent following treatment discontinuation (71). Of platinum drugs, cisplatin is the most emetogenic, causing diarrhea and emesis at high doses in 67% and 70%–80%, respectively (72). In large retrospective study, 86 patients with platinum-based therapy were identified to have suffered from severe colitis and diarrhea (73). About half of these patients also developed abdominal pain and blood or mucus in stool within a median time of 66 days following treatment begin, which lasted for a median range of 20 days. Another 17% had to discontinue treatment due to severe GI symptoms. In a rat model, cisplatin-induced gastrointestinal symptoms were shown to correlate with increased ROS formation, lipid peroxidation, and epithelial apoptosis, leading to damage of mitochondrial DNA and the activation of various signal transduction pathways that culminate in the induction of apoptosis and necrosis (74). Together, these findings indicate that platinum compounds share key cellular stress responses with microtubule-modifying agents, reflecting common vulnerabilities of rapidly proliferating cells.

#### 1.2.3 Anthracyclines

Anthracyclines (doxorubicin, idarubicin) were found to exhibit a multi-pronged cytotoxic mechanism. Their planar ring structure allows these compounds to intercalate between DNA base pairs, physically obstructing the action of enzymes involved in DNA replication and RNA transcription (75–77). They also act as potent “poisons” for the enzyme topoisomerase-II (78, 79). By stabilizing the complex formed between topoisomerase-II and DNA, they prevent the re-ligation of DNA strand breaks that the enzyme normally creates and resolves, leading to an accumulation of permanent DNA damage. Furthermore, the quinone moiety of the anthracycline molecule can undergo redox cycling, a process that generates large quantities of ROS (80, 81). This leads to severe oxidative stress, causing damage to DNA, proteins, and lipids, and further promoting apoptosis. This combination of direct DNA damage, enzyme inhibition, and oxidative stress makes anthracyclines toxic to rapidly dividing cells, including stem/progenitor cells of the intestinal epithelium. Mouse studies showed that although this intestinal population is initially diminished with anthracycline treatment, these cells promote cell lineage expansion upon completion of treatment (82), consistent with regeneration of the epithelium. In conclusion, the intestinal epithelium exhibits robust regenerative capacity following anthracycline treatment, similar to paclitaxel treatment.

#### 1.2.4 Antimetabolites

Antimetabolite chemotherapeutic agents, including 5-fluorouracil (5-FU), the 5-FU prodrug capecitabine, and irinotecan, exert their anticancer effects by disrupting nucleotide metabolism and DNA replication. 5-FU acts through inhibition of thymidylate synthase and incorporation into RNA and DNA, whereas irinotecan inhibits topoisomerase I, thereby impairing proliferation and promoting cell death in rapidly dividing tumor cells (83). This class of chemotherapeutic agents is widely associated with gastrointestinal toxicities characterized by a spectrum of mucosal injury-driven symptoms. These include diarrhea, mucositis and stomatitis, abdominal pain, nausea, vomiting, and, in more severe cases, colitis, ileitis, malabsorption, and weight loss. Among these manifestations, diarrhea represents a frequent and clinically limiting toxicity. In a meta-analysis including 7458 patients treated with 5-FU, 12.6% suffered from grade 3–4 diarrhea, whereas this number was even higher for capecitabine (∼50%) (84). This phenotype was also observed in C57BL/6J mice following 5-FU intraperitoneal (i.p.) injection. Histopathological examinations in this model showed intestinal inflammation, marked by increased cytokines such as TNF-α, IL-1β, IL-6, IL-17A, and IL-22, as well as dysregulation of aquaporins (AQP1, 4, 8, 11) in the upper intestinal tract and colon (85). Use of the TNF inhibitor, etanercept, however suggested that the observed inflammatory response was uncoupled from AQP dysregulation and diarrhea since TNF inhibition did not rescue these phenotypes. Beside diarrhea, capecitabine is associated with abdominal pain, pancreatitis, colitis, ileitis. Histopathological features include epithelial cell apoptosis, crypt damage and loss, mucosal injury, and inflammation (86, 87). The antimetabolite, irinotecan, similarly induces diarrhea and causes weight loss in rodents. In this model, crypt apoptosis appeared within 6 h, and preceded the onset of overt mucosal damage (88). Irinotecan treatment in rats was further associated with reduced tight junction marker expression, suggesting that the mucosal barrier is compromised in this model (89). Besides these effects, antimetabolite drugs have also been linked to intestinal ROS production and mucositis. For instance, irinotecan was shown to induce ROS via the NADPH oxidase, Nox2, which activates caspase-1 and inflammation (90). Also 5-FU induces ROS formation in the small intestine of BALB/C mice (42). These findings underscore that antimetabolite-induced GI toxicity is a coordinated pathological process where early barrier disruption and oxidative stress trigger a range of damaging effects. The persistence of symptoms despite TNF inhibition suggests that effective intervention requires a multi-target approach addressing epithelial stability and ROS signaling rather than inflammation alone.

In conclusion, chemotherapy-induced intestinal toxicities arise from discrete upstream mechanisms but presents with similar phenotypes across multiple drug classes. These shared responses to chemotherapy stressors are examined below in more detail.

### 1.3 Mechanisms governing and amplifying chemotherapy-induced epithelial injury

#### 1.3.1 Chemotherapy-induced epithelial damage extends beyond proliferating crypt cells

The non-specific nature of chemotherapeutic agents, which target proliferating cells, provides a common explanation for epithelial susceptibility. However, this alone does not fully account for the diversity of tissue responses observed across the intestinal tract. While highly proliferative cells, such as intestinal crypt progenitors, are particularly susceptible to chemotherapy-induced damage, accumulating evidence indicates that epithelial injury is not restricted to these cells and can extend to differentiated cell populations. Thus, chemotherapy-induced toxicity reflects not only proliferation-dependent susceptibility, but also context-dependent sensitivity across various epithelial cell states. Epithelial susceptibility, in particular, has been observed not only in the intestine, but also in the skin, which contributes to chemotherapy-induced peripheral neuropathy (CIPN) (91, 92). For example, when paclitaxel conjugated to fluorescein (tubulin tracker) was administered intravenously into larval zebrafish, it accumulated rapidly in skin cells but not neurons, which only selectively showed tubulin tracker fluorescence in some neuron cell bodies (91), hinting at enhanced uptake or retention mechanisms in the skin. Consistent with this, studies in mice further showed that paclitaxel-induced mechanical hypersensitivity is mediated by keratinocytes, whereas optogenetic or chemogenetic keratinocyte inhibition largely alleviated the symptoms during acute and long-term exposure (93). These findings indicate that epithelial cells are not merely passive targets of chemotherapy but actively contribute to the development of treatment-related toxicities. Importantly, the epithelial response to chemotherapeutic agents is not strictly limited to proliferating cells. In larval zebrafish, for instance, most epithelial cells are not actively proliferating, yet these cells rapidly take up paclitaxel and exhibit damage days before axon degeneration commences (91).

Also, vincristine and platinum drugs affect non-mitotic cells. Vincristine was shown to induce apoptosis in interphase cells of mouse small intestinal crypts (59), and cisplatin increases apoptosis not only in proliferating crypt cells but also in differentiated villus enterocytes in the jejunum by 2.3-fold (94). This apoptotic response was attenuated by folate, riboflavin, and a multivitamin mixture lacking α-tocopherol. Notably, unlike the other vitamins tested, α-tocopherol did not protect differentiated villus cells in the presence of cisplatin but reduced apoptosis in proliferating crypts, emphasizing that mitotic and differentiated epithelial compartments respond distinctly to antimitotic cues, with proliferative crypt cells being more amenable to interventional rescue than differentiated villus cells.

The effects of chemotherapeutic agents on non-proliferating cells extends beyond epithelial tissues. The best example is that of non-proliferating neurons, which show degenerate in culture and *in vivo* when exposed to chemotherapeutic agents (95–97). Cardiomyocytes, which have a highly limited proliferative capacity in adults, were shown to be particularly susceptible to anthracycline-induced damage. For example, doxorubicin induces mitochondrial dysfunction and DNA damage through ROS generation and topoisomerase IIβ–mediated mechanisms (98). In the intestine, lineage-tracing experiments using BrdU pulse-labeling demonstrated that antimetabolite chemotherapy drugs, like cytosine arabinoside, rapidly induce apoptosis in S-phase cells within the crypts, indicating that some progenitors die almost immediately after drug exposure rather than undergoing mitosis first (99).

Together, these findings support a model in which chemotherapy-induced epithelial injury occurs across multiple cellular states, affecting both proliferating and non-proliferating cells within the intestine. While proliferative cells remain highly susceptible to rapid damage, differentiated cells can also be damaged. Thus, chemotherapy-induced toxicity may be best understood not solely as a proliferation-dependent toxicity but one that is shaped by chemotherapy regimens, cell proliferation, cellular state, and the activation of stress response pathways, such as through ROS formation. Together, these factors collectively determine the extent and nature of tissue injury.

#### 1.3.2 ROS-driven inflammatory signaling

A key mechanism by which chemotherapeutic agents inflict cellular damage is through the generation of ROS, such as superoxide radicals and hydrogen peroxide (H_2_O_2_). The overproduction of these molecules overwhelms the endogenous antioxidant defense systems of cells, leading to oxidative stress (100). This redox imbalance triggers lipid peroxidation, protein oxidation, and DNA damage. It also activates stress-responsive signaling pathways, such as p53, TNF, NF-κB, and MAPKs (101, 102). In the intestinal epithelium, oxidative stress contributes to crypt cell apoptosis and villus atrophy (94, 102, 103), as well as compromised tight-junction integrity, ultimately impairing the mucosal barrier (104), establishing oxidative stress as both an initiating stress signal and a key driver of injury propagation via inflammation.

The initial epithelial damage triggers intracellular inflammatory signaling pathways (105–107). This response is amplified by oxidative stress and microbial components that arise from barrier disruption. A central pathway in this response is the Nuclear Factor-kappa B (NF-κB) pathway (105, 108). Under normal conditions, NF-κB is held inactive in the cytoplasm. Upon stimulation by danger signals, a cascade is initiated that leads to the degradation of its inhibitor, allowing NF-κB to translocate to the nucleus. Once in the nucleus, NF-κB acts as a master transcription factor, binding to the promoter regions of a vast array of genes involved in the inflammatory response. This results in the rapid and robust production and secretion of pro-inflammatory cytokines (109). Whether oxidative stress acts as a primary driver of downstream pathology or reflects a secondary response to epithelial injury and inflammation remains to be fully resolved.

Within the context of CIGT, several cytokines are considered of central importance in mucositis induced by chemotherapy. These include Tumor Necrosis Factor-alpha (TNF-α), Interleukin-1 beta (IL-1β), and Interleukin-6 (IL-6) (110, 111). Assessment of intestinal mucositis in mice i.p. injected with 5-FU significantly induced these three cytokines in the small intestine and colon in comparison with a saline control group, whereby *Streptococcus thermophilus* ST4 treatment showed protective effects (112). Another study in a murine model of irinotecan (CPT-11)-induced intestinal mucositis showed the upregulation of *Nrf2*, *NF-κB*, *TNF-α*, *IL-1β*, *IL-6*, *IL-17*, *IL-10* in the duodenum. This upregulation was reduced with antioxidants, such as glutathione and malvidin (111), suggesting ROS as an upstream regulator. Interestingly, particularly malvidin improved biochemical and gene expression profiles, but it failed to prevent CPT-11 induced weight loss or colonic tissue injury, indicating that targeting oxidative stress and cytokine pathways alone does not suffice to preserve mucosal integrity and that multiple pathways are activated and responsible for various symptoms. Cytokines are released by both damaged epithelial cells and activated resident immune cells, such as macrophages, creating a strong inflammatory milieu within the gut mucosa (16, 113), which is a key driver of apoptosis (114). TNF-α, for example, can directly induce apoptosis in intestinal epithelial cells together with IL-1β (115, 116), promoting breakdown of tight junctions that compromises barrier integrity (111, 117), further promoting a cycle of inflammation and tissue damage. It remains unclear to what extent inflammatory signaling contributes to symptom generation in acute versus delayed CIGT, and whether it acts as a primary driver or a secondary response to epithelial injury.

Inflammatory cytokines such as TNF-α and IL-1β also induce the expression and activation of matrix metalloproteinases (MMPs), which degrade extracellular matrix components and weaken epithelial barrier integrity. MMPs are zinc-dependent endopeptidases belonging to the gelatinase B family of enzymes, whose primary function is the degradation of components of the extracellular matrix (ECM), including type I, II, IV and V collagens, which are major constituents of basement membranes and ECM (118). MMPs are secreted in their inactive pro-form, primarily by infiltrating immune cells like neutrophils and macrophages, in response to inflammatory stimuli such as TNF-α and IL-1β (119, 120). However, also epithelial cells secrete MMPs. MMP expression is often increased under conditions of chemotherapy treatment (92, 121). In preclinical models of chemotherapy-induced GI toxicity, for instance, MMP-2, -3, -9 and -12 levels were associated with inflammatory infiltrate and maximum tissue damage in the jejunum and colon (122). In a rat model for irinotecan treatment, activation of MMP-9 significantly contributed to mucositis pathobiology by enzymatically dismantling the structural scaffold in the mucosa through the degradation of the basement membrane and other ECM components. MMP-9 activity was shown to exacerbate the loss of barrier integrity, facilitate the infiltration of more inflammatory cells, and contribute to the overall tissue remodeling and ulceration characteristics of severe mucositis (123).

While much of the focus has been placed on MMP-2 and MMP-9, emerging evidence highlights MMP-13 as a potentially critical mediator of intestinal injury in inflammatory contexts. Alongside MMP-9, MMP-13 has been identified as a critical mediator in inflammatory intestinal diseases that share pathological features with CIGT, such as inflammatory bowel disease (IBD) (124) and sepsis (124, 125). Like other MMPs, MMP-13 is a zinc-dependent enzyme capable of degrading extracellular matrix components, with a preference for cleaving type II collagen, but also acting on types I and III (118). Its expression is typically low under normal physiological conditions and upregulated during inflammatory conditions, including chemotherapy treatment (97, 126–128). A crucial pathogenic role for MMP-13 in gut injury stems from its ability to act as a “sheddase” for TNF-α. TNF-α is produced as an inactive membrane-bound precursor (pro-TNF), which must be cleaved to release the soluble, biologically active form that drives inflammation. While the enzyme TACE is the primary TNF sheddase, studies in animal models of sepsis and colitis have demonstrated that MMP-13 can also perform this cleavage (125). In these models, MMP-13-deficient mice were strongly protected from gut injury as they exhibited reduced intestinal permeability, less depletion of mucus-producing goblet cells, and decreased endoplasmic reticulum stress. This protection was directly linked to lower levels of active, soluble TNF in the gut, suggesting that MMP-13 is a key contributor to inflammation and barrier damage via TNF regulation. Intriguingly, MMP-13 was shown to play a critical role in promoting CIPN via epithelial upregulation (92, 121). Thus, MMP-13 may be a potential therapeutic target for CIGT should its mechanisms be conserved in the chemotherapy-exposed intestine.

#### 1.3.3 The gut microbiome as a modifier and amplifier of toxicity

The gut microbiome, which resembles the complex community of microorganisms residing in the intestinal lumen, plays a critical role in both amplifying and being affected by CIGT. Chemotherapy itself induces profound shifts in the composition and function of this microbial community, a state known as dysbiosis. This shift is not a passive consequence but an active component of the pathogenic cascade. For instance, fecal sample analysis using 16S RNAseq in patients with non-Hodgkin’s lymphoma who received the same myeloablative conditioning regimen, which included high-dose Carmustine (Bis-chloroethylnitrosourea), Etoposide, Aracytine and Melphalan and no other concomitant therapy such as antibiotics, showed enrichment in proteobacteria at the expense of actinobacteria and firmicutes, indicating an increased inflammatory environment (129). How can a dysbiotic microbiome contribute to CIGT? A potential link is the observation that irinotecan promoted microbial components, such as lipopolysaccharide (LPS) to translocate across the intestinal epithelial barrier, which was dependent on the Toll-like receptor (TLR-4). In contrast, TLR-4 knockout mice were protected from irinotecan-induced barrier disruptions (130). Irinotecan treated mice also showed a marked expression of TNF-α, IL-1β, IL-6, and IL-10, suggesting a strong inflammatory response, which was muted in the knockout mice. Microbial components are potent pathogen-associated molecular patterns (PAMPs) that are recognized by pattern recognition receptors, such as TLRs on immune and epithelial cells (131). Their recognition has been shown to trigger the activation of the innate immune system, leading to NF-κB signaling and amplification of these cytokines (132). Thus, barrier disruption and microbial invasion may establish a destructive positive feedback loop, which may explain how an initial, discrete chemotherapy-induced injury may escalate into a more severe, widespread, and self-sustaining inflammatory condition that defines the clinical syndrome of CIGT. The combined effects of proliferation-dependent epithelial damage and ROS-driven stress responses likely contribute to a cascade of events involving inflammatory signaling, extracellular matrix remodeling, and microbiome dysbiosis, collectively driving the progression from localized injury to systemic gastrointestinal toxicity. However, the precise contributions of microbiome changes to CIGT remains incompletely defined and is worth further investigating. In particular, metabolic crosstalk between the gut microbiome and the nervous system in this context has not been clearly established, despite extensive evidence that the gut-brain axis can modulate neural functions through microbial metabolites and signaling pathways. For instance, microbiota can synthesize and respond to 5-HT, as well as other neurotransmitters such as GABA, which influence host neural functions (133). They also produce short-chain fatty acids and modulate tryptophan metabolism, both of which can influence gut-brain signaling. These metabolites have been shown to affect enteroendocrine cells, vagal afferent activity, and immune pathways, and have been implicated in the regulation of visceral pain and gut-brain communication. However, their direct contribution to nausea and vomiting in CIGT have not been established. Clarifying their contributions may reveal new therapeutic opportunities for CIGT.

### 1.4 The mucosal-neural axis: epithelial-to-neuron crosstalk in CIGT

The mucosal-neuronal axis refers to the bidirectional crosstalk between the intestinal epithelium and the nervous system, including DRG afferents, vagal afferents, and enteric neurons. It is well established that epithelial injury and barrier dysfunction activate sensory neurons, while neural signaling in turn modulates epithelial integrity, immune activation, and the local cytokine environment. However, the mechanisms linking epithelial damage to coordinated responses across enteric and extrinsic neural circuits remain incompletely defined in the context of CIGT. We propose that epithelial injury induces a broader metabolic stress response that engages both intrinsic and extrinsic neural pathways, contributing to gastrointestinal dysfunction. In addition, emerging evidence from peripheral neuropathy models suggests that DRG neurons can become intrinsically sensitized by chemotherapeutic agents, raising the possibility that altered DRG activity may also influence gut functions independently of local epithelial injury. This section focuses on the mechanisms linking epithelial injury to neural responses and the role of the immune system in modulating these processes.

The clinical presentation of CIGT, characterized by abdominal pain, nausea, and altered gut motility, cannot be explained by epithelial injury alone, particularly given the clinical efficacy of antiemetic therapies that target neurotransmitter signaling within peripheral and central neural circuits (134). Chemotherapeutic agents perturb neural circuits that regulate gastrointestinal function, implicating both the intrinsic enteric nervous system (ENS) and extrinsic sensory pathways in disease pathogenesis (Table 3). It has been demonstrated that chemotherapy induces structural and functional alterations in enteric neurons and glial cells in preclinical models, leading to impaired enteric neuronal control of gut motility and secretory function (135–137). In parallel, several CIPN models have demonstrated that DRG neurons (the cell bodies of primary spinal sensory afferents) become hyperexcitable and exhibit altered electrophysiological properties in response to chemotherapeutic agents, such as paclitaxel (97). Since DRG neurons innervate the intestinal wall, they could directly signal to the epithelium to reduce the inflammatory environment, such as during epithelial recovery. It is well established that DRG neurons communicate with epithelial cells, and exert protective effects. For instance, Nav1.8^+^ CGRP^+^ nociceptors innervating the gut direct goblet cells to increase mucus secretion, which provide protective effects to the mucosa (138). Direct evidence for DRG involvement in CIGT is however lacking. It is further possible that chemotherapy exposure induces DRG degeneration, which may compromise the recovery, which remains to be investigated. DRG neurons may also integrate pathological signals arising from intestinal injury and relay this aberrant sensory input to the central nervous system. This is has been shown in the context of CIPN where paclitaxel and related chemotherapeutics induce DRG neuron hyperexcitability and spontaneous firing. For instance, whole-cell patch-clamp recordings of DRG neurons *in vitro* showed that paclitaxel exposure for 24 or 48 h induced spontaneous depolarizing fluctuations (DSFs) and increased excitability without eliciting ectopic action potential activity. Evidence of oxidative stress and mitochondrial toxicity also emerged after 48 h of paclitaxel exposure (139). Therefore, studies that examine DRG signaling to the intestinal epithelium and to the brain in the context of CIGT will help establish their particular role in contributing to this condition.

**TABLE 3:** Key players involved in mucosal and neuronal damage.

Pathogenic process	Key molecular player(s)	Primary cellular source(s)	Primary role in CIGT
Epithelial barrier disruption	Claudins, occludin, ZO-1, E-cadherin	Intestinal epithelial cells	Loss of paracellular seal, increased permeability
Oxidative stress	4-Hydroxynonenal (4-HNE)	Peroxidized cell membranes	Covalent modification of proteins, apoptosis induction, inflammation
Inflammatory signaling	NF-κB, TNF-α, IL-1β, IL-6	Epithelial cells, immune cells (e.g., macrophages)	Transcription of pro-inflammatory genes, amplification of inflammation, induction of apoptosis
Matrix remodeling	Matrix Metalloproteinase-9 (MMP-9)	Neutrophils, macrophages	Degradation of basement membrane and extracellular matrix, exacerbation of barrier loss
Neuronal sensitization	Serotonin	Damaged epithelial cells, enterochromaffin cells	Activation and sensitization of enteric and dorsal root ganglion neurons, leading to pain, and nausea and vomiting

#### 1.4.1 Pathophysiology of chemotherapy-induced enteric neuron and visceral hypersensitivity

Multiple chemotherapeutic agents exert direct neurotoxic effects on the ENS. Oxaliplatin treatment in mice, for instance, induces a significant loss and the remodeling of myenteric neurons, accompanied by changes in neurochemical coding and disruption of colonic motility (137). Notably, these neuronal changes arise in parallel with epithelial injury but can be detected even in the absence of extensive epithelial damage. Thus chemotherapeutic stress may directly perturb enteric neural integrity while epithelial damage may further amplify dysfunction. Alternatively, subtle epithelial perturbations may be sufficient to drive neuronal dysfunction, consistent with observations in other epithelial-neuronal interfaces, such as the skin epithelium. Further studies demonstrated that oxaliplatin alters both neuronal and glial populations within the myenteric plexus, leading to reductions in nerve fiber density and enteric glial cells. These changes are associated with impaired neuromuscular transmission and dysregulated smooth muscle contractility (140). Together, these findings establish a direct mechanistic link between chemotherapy-induced enteric neurotoxicity and the motility disturbances that define CIGT.

Preclinical studies further demonstrated that acute paclitaxel administration elicits visceral pain (nociception), as well as mechanical and thermal hypersensitivity through TRPV1-dependent sensory pathways. In rodents, a single systemic dose of paclitaxel (1 mg/kg) induced rapid visceral nociceptive responses and acute hypersensitivity, effects that are markedly attenuated by pharmacological TRPV1 inhibition and in TRPV1-deficient mice (141). Population-based studies of cancer survivors indicate that exposure to chemotherapy is also linked to a higher burden of chronic gastrointestinal complaints, including abdominal discomfort and bloating, which are associated with reduced physical and mental wellbeing. Predominantly breast cancer survivors were included in this study, pointing to a potential contribution of taxane-based regimens, such as paclitaxel, to gut-brain axis disturbances (142).

**FIGURE 3:**
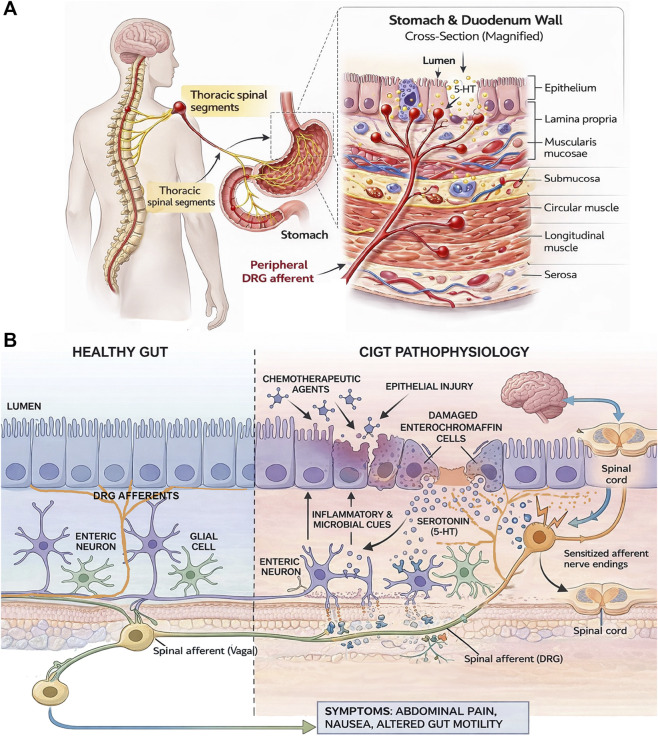
Integrated epithelial-neural mechanisms underlying CIGT. This illustration depicts the coordinated pathophysiological cascade linking chemotherapy-induced epithelial injury to neural dysfunction and clinical symptoms. Established epithelial injury pathways, including oxidative stress, barrier disruption, and dysregulated 5-HT signaling, are integrated with emerging and less well-defined mechanisms linking epithelial damage to enteric and extrinsic neural dysfunction. In particular, the pathways connecting barrier disruption, microbial and inflammatory signals, and the sensitization of DRG afferents represent areas where direct experimental evidence in CIGT remains limited and are therefore presented as an integrated model. **(A)** The intestinal mucosa comprises the epithelium, lamina propria, and muscularis mucosae. Beneath the mucosa lies the submucosa, followed by the muscularis externa (circular and longitudinal muscle layers) and the serosa. A single DRG afferent (red) enters from the serosal side and branches into multiple compartments, forming sensory terminals (represented by red bulbs) within the circular muscle layer, submucosa, and lamina propria of the mucosa. Mucosal terminals are positioned in close proximity to epithelial and enterochromaffin (EC) cells, allowing for paracrine communication. This organization enables spinal afferents to detect mechanical, chemical, and inflammatory signals arising from distinct gut wall layers and transmit sensory information to the central nervous system. **(B)** The left panel depicts a healthy gut with intact epithelial barrier function and normal enteric and extrinsic neural architecture. The right panel shows CIGT pathophysiology, where chemotherapeutic agents induce epithelial damage through mechanisms including ROS generation and inflammatory signaling, resulting in barrier disruption. This injury affects enterochromaffin cells and promotes dysregulated 5-HT release. In parallel, microbial and inflammatory signals arising from barrier compromise are proposed to further amplify neural activation. Together, these signals are hypothesized to disrupt enteric neuron and glial cell function and contribute to sensitization of DRG afferents. Aberrant signals due to neuronal hyperexcitation are transmitted to the central nervous system, leading to pain, nausea, vomiting, visceral hypersensitivity, and altered gut function, characteristic of CIGT.

Beyond direct neuronal injury, chemotherapy-induced epithelial stress dependent inflammatory and oxidative signaling within the gut wall can further modulate neuronal function. Oxaliplatin treatment, for instance, has been shown to induce local inflammatory responses and microbial alterations that coincide with enteric neuronal remodeling and functional deficits (140). This suggests that epithelial and microbial changes may influence neuronal remodeling. Such changes are consistent with, and may contribute to, sensitization gut-innervating sensory pathways, which has been proposed as a mechanistic basis for abdominal pain and cramping experienced during CIGT. This type of sensitization is reflected in colorectal distension models, where increased sensitivity of gut-innervating sensory neurons leads to exaggerated pain-related motor responses, indicating visceral hypersensitivity (143). In the context of chemotherapy-induced epithelial and enteric neuronal injury, such sensitization would be expected to render normally innocuous physiological stimuli painful, producing allodynia and hyperalgesia. In conclusion, chemotherapeutic agents disrupt epithelial integrity and also directly damage enteric neurons, while potentially promoting inflammatory and microbial crosstalk that further sensitizes neural pathways (Figure 3).

#### 1.4.2 Serotonin is a key mediator of neuronal sensitization

The gut epithelium is the primary source of serotonin for the body, which is synthesized and stored in specialized enterochromaffin cells (144). Mucosal damage during CIGT leads to the release of 5-HT into the gut wall (5, 145, 146). Serotonin then acts on a wide variety of 5-HT receptor subtypes expressed on both enteric neurons and extrinsic sensory afferent nerves that project to the spinal cord (147). Mechanistically, docking studies suggest that certain antineoplastic agents, including cisplatin, dacarbazine and nitrogen mustard, may compete with serotonin to directly bind to the 5-HT3 receptor and stimulate an emetic response (148). This signaling ultimately affects gut motility, secretion, and the perception of pain, establishing 5-HT as a critical sensitizing agent in chemotherapy-induced distress (149, 150). A direct link between cisplatin and serotonin dependent vagal activation was demonstrated using electrophysiological recordings in rats where cisplatin administration triggered an immediate increase in vagal afferent firing. This response was abolished by the 5-HT3 receptor antagonist, Y-25130 (151). While serotonergic signaling is well established in acute CINV, its role in mediating sustained gastrointestinal dysfunction in CIGT remains less clearly defined.

#### 1.4.3 Sensitization of DRG afferents

The primary sensory neurons that transmit information, including pain signals, from the gut to the central nervous system have their cell bodies located in the dorsal root ganglia (DRG), which lie just outside the spinal cord (152, 153) (Figure 3A). These neurons are a principal target of chemotherapy-induced neurotoxicity (154, 155). The damage occurs through two convergent pathways. First, chemotherapeutic agents like paclitaxel and cisplatin can cross the blood-nerve barrier (156, 157) and directly damage the DRG neurons. This could in principle influence the intestinal mucosa which is innervated by peripheral axons of some DRGs. Known effects on DRG neurons that have been elucidated in the context of CIPN are mitochondrial dysfunction (158, 159), pathological alterations in ion channel expression and function (160, 161), including dysregulation of intracellular Ca^2+^ release channels and calcium homeostasis, and axonal degeneration (162, 163). Second, the inflammatory and neuro-sensitizing mediators released from the damaged gut (TNF-α, IL-6, 5-HT) enter the systemic circulation and may reach the DRG (164–166), leading to a secondary inflammatory response within the ganglia themselves, characterized by the infiltration of immune cells like macrophages (155). The combination of direct cytotoxic insult and indirect inflammatory sensitization renders the DRG neurons hyperexcitable (161). This state of central sensitization within the DRG is a crucial mechanism that underlies the chronic, burning pain and hypersensitivity in the extremities known for CIPN (155, 161) and may act similarly in the gut leading to CIGT. In addition, the skin epithelium has been shown to overexpress MMP-13 and produce excessive ROS in the presence of chemotherapeutic agents like paclitaxel, which contributes to axon degeneration in CIPN. The intestinal mucosa upregulates MMP-13 in response to inflammatory cues like LPS (124), and thus MMP-13 or other MMPs may contribute to CIGT via DRG axon damage. The extent to which this mechanism is involved in abdominal pain and visceral hypersensitivity or nausea and vomiting following chemotherapy remains to be explored.

#### 1.4.4 Evidence for an integrated neuro-epithelial axis

Although no single study presented explicitly connects DRG sensitization to chemotherapy-induced intestinal injury, a DRG-to-neuron crosstalk hypothesis in CIGT is a robust and compelling inference drawn from the convergence of strong evidence from highly analogous and mechanistically overlapping fields. 1) Inflammatory gut models replicate a state of intense gut inflammation similar to CIGT, directly demonstrating that inflammatory mediators like TNF-α sensitize colon-innervating DRG neurons via modulation of ATP-sensitive P2X3 receptors ((165–167). Research on irritable bowel syndrome (IBS) has established a firm role for epithelial-derived serotonin in driving visceral pain and hypersensitivity through its action on sensory afferents (168–170). Finally, studies focusing on the somatic (non-visceral) neurotoxicity of chemotherapy have implicated epithelial damage as a key driver of DRG axon degeneration and pain (97, 171, 172). Based on the pathophysiology of CIGT, we hypothesize that a similar environment exists in the gut mucosa, characterized by epithelial damage, inflammation, and neurotoxicity, in which serotonin is released from ECs and exerts its sensitizing effects on sensory neurons. This crosstalk hypothesis is a logical consequence from the provided evidence of mechanisms leading to intestinal pathology.

This integrated view also suggests that CIGT and CIPN may not represent entirely separate toxicities. Instead, they appear to be mechanistic cousins, representing the visceral and somatic manifestations of a common underlying pathology: chemotherapy-induced neuroinflammation. The process begins with direct damage to both the gut epithelium (initiating CIGT) and the DRG neurons (initiating CIPN). The subsequent “leaky gut” caused in CIGT (104, 173, 174) then can become a systemic source of pro-inflammatory and pro-nociceptive signals produced by altered gut microbes. These signals then travel to the DRG leading to neuropathic pain (175, 176). Together, these observations suggest that gut-specific effects of chemotherapeutic agents may not only participate in visceral pain and nausea and vomiting, but may also amplify neuronal sensitization and drive CIPN symptoms (177, 178). This model may help explain the frequent and debilitating clinical overlap in patients who suffer from both severe gastrointestinal distress and peripheral neuropathy. It also raises the intriguing therapeutic possibility that interventions aimed at restoring gut barrier integrity could have beneficial effects on the symptoms of peripheral neuropathy and vice versa.

#### 1.4.5 Future research on mucosal-neural crosstalk

Although serotonin has long been recognized as a central mediator of gut-neural communication, its precise role in chemotherapy-induced gastrointestinal toxicity (CIGT) remains incompletely defined. *In vivo* studies that directly link EC-derived serotonin to changes in visceral afferent firing during chemotherapy are still limited. While serotonin is well established as a driver of visceral sensory signaling in IBS, where it contributes to hypersensitivity, nausea, and motility changes via 5-HT_3_ and 5-HT_4_ receptors (179, 180), its causal contribution and mechanistic positioning in chemotherapy-induced mucosal injury may differ in magnitude, timing, and downstream integration.

A major unresolved question is how serotonergic signaling interfaces with other candidate epithelial- or immune-linked mediators that can sensitize sensory neurons in gastrointestinal inflammation more broadly. For example, purinergic ATP-P2X3 signaling and NGF-dependent mechanisms have been implicated in neuronal sensitization in diverse pain and inflammatory models, but their relevance, temporal recruitment, and symptom-specific contributions in CIGT remain to be established. Determining whether these pathways act independently of serotonin, converge on shared excitability pathways, or operate in a hierarchical manner to drive distinct outcomes such as visceral pain, nausea, and dysmotility would help to clarify the underlying mechanisms of chemotherapy-induced vomiting and nausea. Chemotherapy induces the expression of various MMPs, including *MMP2, MMP3, and MMP9* (181) in the oral mucosa, presumably mediating remodeling of the mucosal microenvironment via changes in the extracellular matrix and barrier properties. Defining the relative contributions and mechanistic integration of these MMPs and others, such as MMP-13 in neuronal damage could be useful in determining mechanisms that underlie neuronal activation. Peripheral sensory afferents with origin in the DRG arborize within the mucosa and closely appose epithelial and ECs, positioning them to respond dynamically to local microenvironmental changes (Figure 3) (182). It is conceivable that chemotherapy-induced epithelial stress alters this neuroepithelial interface through mechanisms that include extracellular matrix remodeling, and this could influence DRG terminal accessibility, branching architecture, or local receptor exposure. Such remodeling might also indirectly shape serotonergic signaling by altering the diffusion, concentration gradients, or receptor microdomains associated with 5-HT_3_ and 5-HT_4_ pathways. Whether extracellular matrix remodeling functions upstream of serotonergic activation, operates in parallel, or independently regulates afferent sensitization in CIGT remains unknown. Direct *in vivo* studies examining DRG terminal structure, neuronal firing, and mucosal MMP activity and their involvement will be required to define the contribution of these interactions to symptoms such as nausea, visceral pain, and dysmotility. A critical next step is therefore to analyze these potential links. Other approaches could involve preclinical studies that use specific pharmacological tools, such as P2X3 receptor antagonists, or anti-NGF neutralizing antibodies, to determine if blocking these individual pathways can alleviate specific CIGT symptoms like visceral pain, nausea, or dysmotility. Furthermore, the communication is likely bidirectional; the nervous system can also influence mucosal inflammation and repair given the evidence from cancer studies (183–185). Investigating chemotherapy-induced enteric neuropathy and axon degeneration and how this might contribute to the mucosal ability to heal or regulate local inflammation is another important and underexplored area of research. A deeper understanding of this complex crosstalk is essential for developing novel therapies that can target the neurological symptoms of CIGT at their source.

### 1.5 Current management of CIGT and its limitations

In light of the mechanisms described above, current management strategies for CIGT remain largely supportive and do not directly target the underlying drivers of epithelial injury, inflammation, and neuro-epithelial signaling. These aim at symptom management rather than preventing the underlying pathology, and are often insufficient to prevent dose-limiting toxicities.

#### 1.5.1 Symptom-specific CIGT management

The approach to treatment is typically tailored to the specific symptoms of the patient but follows specific regimens (Table 4). *Nausea and Vomiting (CINV):* This is the most successfully managed aspect of CIGT, largely due to the development of mechanism-based therapies. The understanding that chemotherapy triggers serotonin release from enterochromaffin cells in the gut led to the design of 5-HT3 receptor antagonists (e.g., ondansetron) (5, 24). These are the cornerstone of CINV prevention and are highly effective, especially for acute emesis. Prophylactic regimens for moderate-to-high risk chemotherapy now typically involve a multi-drug approach, combining a 5-HT3 antagonist with a neurokinin-1 (NK-1) receptor antagonist (e.g., aprepitant) and a corticosteroid (e.g., dexamethasone) (134, 186). *Diarrhea (CID):* Standard management focuses on fluid and electrolyte replacement to prevent dehydration, along with the use of anti-motility agents, with loperamide being the first-line therapy. For diarrhea that is severe or refractory to loperamide, the somatostatin analog octreotide may be used to reduce gastrointestinal secretions (187, 188). In irinotecan-associated diarrhea, budesonide has shown activity in some refractory-treatment reports, though randomized prevention data have been negative (189, 190). *Mucositis (oral and intestinal):* Treatment for mucositis is primarily palliative. For oral mucositis, care focuses on maintaining oral hygiene with gentle brushing and saline rinses, managing pain with topical anesthetics (“magic mouthwash”) and systemic analgesics, and maintaining nutrition with a soft, bland diet. Cryotherapy (sucking on ice chips during infusion of certain chemotherapies) can reduce the severity of oral mucositis (191). Photobiomodulation (low-level laser therapy) is used in specific settings to prevent oral mucositis and can reduce mucositis-associated pain (191). To date, palifermin (keratinocyte growth factor) is FDA-approved to decrease the incidence and duration of severe oral mucositis in patients with hematologic malignancies receiving myelotoxic therapy with autologous hematopoietic stem cell support (192). Management of intestinal mucositis involves treating the symptoms it causes, such as diarrhea and abdominal pain. *Abdominal pain:* Pain associated with CIGT is managed according to the general principles of cancer pain management, often following the World Health Organization (WHO) three-step analgesic ladder (193). This approach begins with non-opioid analgesics like acetaminophen and NSAIDs, escalating to weak and then strong opioids as needed. For neuropathic pain components, adjuvant medications such as anticonvulsants or antidepressants may be added.

**TABLE 4:** Antiemetic drug guidelines.

Antiemetic class	Typical role in guidelines (HEC/MEC/LEC^a^)	Key practical notes
5-HT_3_ receptor antagonists (ondansetron, granisetron, palonosetron, ± extended-release formulations)	Foundation for acute-phase prophylaxis in most MEC and HEC regimens; typically given day 1	Best for acute emesis; less effective alone for delayed symptoms. Palonosetron is commonly preferred when delayed control is a concern (longer half-life; higher receptor affinity) and is frequently used in MEC/selected HEC regimens
NK_1_ receptor antagonists (*aprepitant/fosaprepitant, netupitant, rolapitant*)	Component of standard prophylaxis for HEC; also used for carboplatin-based (higher-risk MEC) in many guideline algorithms. Always combined with a 5-HT_3_ agent + dexamethasone (and often olanzapine for HEC)	Improves delayed-phase and overall control. Netupitant is available as a fixed combination with palonosetron (NEPA)
Corticosteroids (dexamethasone)	Core component of prophylaxis for MEC and HEC, used in combination with a 5-HT_3_ antagonist and, for HEC (and selected higher-risk MEC), an NK1 antagonist ± olanzapine	Enhances efficacy of other antiemetics; dosing duration varies by regimen. Some NK1 receptor antagonists require dexamethasone dose adjustment (often reduction) due to CYP3A4 interactions
Olanzapine	Commonly recommended as part of HEC prophylaxis (often 4-drug prophylaxis: Olanzapine + NK1 + 5-HT_3_ + dexamethasone). Also used for breakthrough CINV.	Particularly helpful for nausea control (both acute and delayed). Main limitation is sedation (dose-dependent); 5 mg is frequently used to improve tolerability in practice
Dopamine antagonists/anti-psychotics (D_2_ pathway) (metoclopramide, prochlorperazine, haloperidol)	Primarily breakthrough/rescue therapy; not preferred for routine prophylaxis in HEC/MEC when modern regimens are available	Useful add-ons when symptoms break through; monitor for extrapyramidal symptoms and sedation (metoclopramide also has pro-kinetic effects)
Cannabinoids (dronabinol, nabilone)	Considered for refractory CINV when standard prophylaxis and rescue options fail; not first-line prophylaxis	Can reduce nausea/vomiting in some patients but limited by CNS side effects (dizziness, dysphoria, sedation) and variability in response
Fixed combinations (NEPA) (netupitant + palonosetron)	A simplified way to deliver NK1 + 5-HT_3_ backbone (typically with dexamethasone; ± olanzapine for HEC)	Convenience/adherence advantage; used across multiple cycles with guideline-consistent regimens
Benzodiazepines (lorazepam)	Adjunct for anticipatory nausea/anxiety and breakthrough symptoms; not primary antiemetic prophylaxis	Sedation and dependence risk

^a^ HEC/MEC/LEC: Highly/Moderately/Low emetogenic chemotherapy. Phases: acute (0–24 h), delayed (24–120 h), and overall (0–120 h).

#### 1.5.2 Overall effectiveness, translational implications, and unmet needs

Current therapeutic strategies for CIGT remain largely symptom-based and predominantly target neural signaling pathways (Table 5). Antiemetic regimens, including 5-HT_3_ receptor antagonists (e.g., ondansetron) and NK1 receptor inhibitors (e.g., aprepitant), represent a major success in supportive oncology care and effectively control acute nausea and vomiting in a substantial proportion of patients (194–196). Additional agents such as dexamethasone and olanzapine further enhance control of emesis through central mechanisms. However, within the framework of the mucosal-neural axis, these therapies primarily modulate downstream neural circuits and do not address the initiating epithelial injury or its downstream inflammatory and structural consequences. This disconnect has important clinical implications. While acute CINV is relatively well controlled, other manifestations of CIGT, including diarrhea, mucositis, abdominal pain, and dysmotility, remain inadequately managed. Current interventions for these symptoms are largely reactive and palliative. Loperamide is the standard first-line therapy for chemotherapy-induced diarrhea, and octreotide is used in severe or refractory cases, but these approaches aim to reduce stool output and secretion rather than restore epithelial barrier integrity or prevent mucosal injury (197, 198). For mucositis, most interventions remain supportive, focusing on oral hygiene, pain control, and nutritional maintenance, although palifermin provides proof of principle that epithelial-directed therapy is feasible in selected settings. Pain management similarly follows general analgesic principles and does not specifically target the mechanisms driving chemotherapy-induced mucosal and neural dysfunction. Emerging preclinical evidence suggests that epithelial injury is not merely a passive consequence of chemotherapy but an active driver of disease progression through barrier disruption, oxidative stress, and extracellular matrix remodeling. These processes may promote microbial translocation, sustain inflammatory signaling, and amplify neural sensitization. However, their clinical relevance remains incompletely established, and there are currently no approved therapies that directly target epithelial integrity, barrier restoration, or matrix remodeling in CIGT. From a translational perspective, this identifies several mechanistically defined therapeutic opportunities, which could be further explored.

**TABLE 5:** The effectiveness of antiemetics against CINV.

Drug class	Examples	Mechanism of action	Acute control*	Delayed control*	Key limitations
5-HT_3_ receptor antagonists (1st gen)	Ondansetron, granisetron, dolasetron	Block 5-HT_3_ signaling on vagal afferents and within brainstem emetic circuitry	High (for vomiting)	Low-moderate	Less effective for delayed phase and for nausea than for emesis; usually needs dexamethasone ± NK1 (and/or olanzapine in HEC)
5-HT_3_ receptor antagonists (2nd gen)	Palonosetron	Same pathway as above; longer half-life and pharmacologic features associated with improved delayed control vs. some 1st-gen agents	High	Moderate	Still incomplete for nausea in higher-risk regimens; best used as part of combination prophylaxis
NK1 receptor antagonists	Aprepitant, fosaprepitant, netupitant, rolapitant	Block substance P/NK1 signaling in central emetic pathways (brainstem)	Moderate (incremental benefit)	High	Greatest impact is delayed/overall control; used with 5-HT_3 + dexamethasone (± olanzapine for HEC). Drug–drug interactions for some agents (CYP3A4)
Corticosteroids	Dexamethasone	Multifactorial antiemetic effect; potentiates 5-HT_3_/NK1 regimens	Moderate (as adjunct)	Moderate (as adjunct)	Not preferred as monotherapy except low-risk settings; adverse effects and regimen-dependent dosing duration; dose adjustment may be needed with some NK1 agents (CYP3A4)
Olanzapine	Olanzapine	Antagonizes multiple receptors relevant to nausea/vomiting (dopaminergic, serotonergic, histaminergic)	High (notably nausea)	High (notably nausea)	Sedation/metabolic effects; dose often lowered (e.g., 5 mg) to improve tolerability; included in many HEC prophylaxis regimens and used for breakthrough
Dopamine antagonists	Metoclopramide, prochlorperazine	Block D_2_ signaling in chemoreceptor trigger zone; metoclopramide also pro-kinetic	Low–Moderate (mostly rescue)	Low	Primarily for breakthrough/rescue; EPS, sedation, QT concerns (agent-dependent) limit prophylactic use
Cannabinoids	Dronabinol, nabilone	CB1 receptor agonism/modulation of central emetic signaling	Low–Moderate (refractory)	Low–Moderate (refractory)	Reserved for refractory CINV; CNS side effects (dizziness, dysphoria, sedation) and variable response
Fixed combo (NEPA)	Netupitant + palonosetron	Combined NK1 + 5-HT_3_ blockade	High	High–Moderate	Convenience/adherence advantage; still typically paired with dexamethasone (± olanzapine in HEC); regimen context determines effectiveness

Acute control: Effectiveness of specified drug within the first 0–24 hours. Delayed control: Effectiveness of specified drug within 24–120 hours. (HEC/MEC), regimen (e.g., cisplatin, AC, carboplatin), and patient risk factors.

##### 1.5.2.1 Epithelial barrier integrity and regeneration

Clinical use of palifermin demonstrates that epithelial-directed therapies are feasible. Additional approaches under investigation include glutamine supplementation, tight junction stabilizers, and microbiome-directed strategies (e.g., probiotics or fecal microbiota transplantation), although results remain variable and context-dependent.

##### 1.5.2.2 Oxidative stress and mitochondrial injury

Reactive oxygen species are increasingly implicated in epithelial damage. Antioxidants such as N-acetylcysteine, mitochondrial-targeted ROS modulators, and superoxide dismutase mimetics show protective effects in preclinical models, but clinical validation is limited.

##### 1.5.2.3 Extracellular matrix remodeling and barrier breakdown

Matrix metalloproteinases (e.g., MMP-2, -9, -13) contribute to structural degradation of the epithelial barrier and may represent a critical transition point from reversible injury to sustained pathology. While prior broad-spectrum MMP inhibitors were limited by toxicity, selective targeting of specific MMPs represents a promising but as yet unexplored strategy in CIGT.

##### 1.5.2.4 Inflammatory amplification

Cytokine-driven pathways (e.g., TNF-α, IL-1β, IL-6) sustain mucosal inflammation. Biologic therapies targeting these pathways are effective in other inflammatory conditions but remain largely untested in CIGT and may be constrained by immunosuppressive risks in cancer patients.

##### 1.5.2.5 Mucosal-neural signaling and visceral hypersensitivity

Current antiemetics effectively target central and peripheral neural pathways involved in nausea and vomiting. Additional neuromodulators, including gabapentinoids and tricyclic antidepressants, may alleviate pain and hypersensitivity but do not address upstream epithelial drivers.

Taken together, current clinical management is effective for downstream neural symptoms, while upstream epithelial damage, barrier dysfunction, and inflammatory amplification remain areas in which treatment could be improved. A key translational opportunity lies in combination strategies that integrate epithelial-protective or barrier-restoring therapies with established neuro-targeted regimens. Targeting critical upstream processes, particularly epithelial stress responses and extracellular matrix remodeling, as well as junction proteins may prevent the initiation and amplification of mucosal-neural signaling. However, progress in this area is limited by the lack of validated biomarkers linking epithelial injury to functional outcomes, and by a scarcity of clinical trials designed to test mechanism-based interventions in CIGT.

### 1.6 Discussion

**FIGURE 4:**
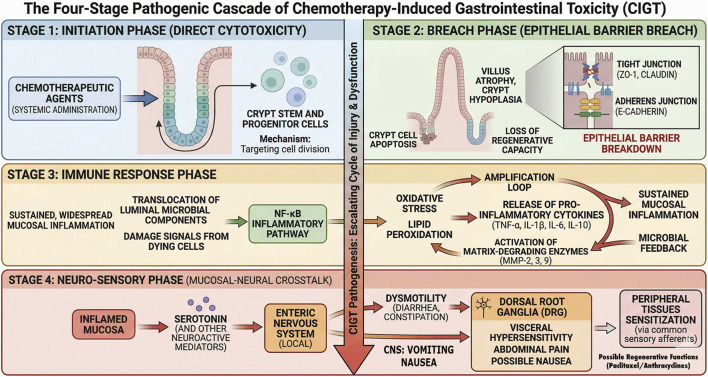
Four stage model of the pathogenesis and clinical sequelae of CIGT. Chemotherapy administration induces direct cytotoxic injury to the gastrointestinal mucosa, leading to mucositis characterized by inflammation. This primary damage is associated with a complex interplay of pathogenic factors, including gut microbiota dysbiosis, dysregulated inflammatory responses, oxidative stress, and altered neurotransmitter signaling. While epithelial injury, oxidative stress, and serotonin-mediated signaling are well established, the integration of microbial, inflammatory, and neural pathways, particularly their roles in enteric and DRG neuron sensitization, remains incompletely defined and is presented here as a conceptual framework. These mechanisms are proposed to contribute to activation of enteric and extrinsic neural pathways, propagating signals to the central and peripheral nervous systems. The resulting physiological disruption manifests as a constellation of clinical symptoms, including nausea, vomiting, diarrhea, and abdominal pain.

The comprehensive analysis of the pathobiology of CIGT reveals a complex, multi-stage process that extends from initial molecular damage to systemic neuro-immune dysregulation. Integrating this knowledge into a cohesive model not only clarifies the disease process but also illuminates potential avenues for much-needed therapeutic intervention. The pathogenesis of CIGT can be conceptualized as a four-stage cascade, where each stage builds upon the last, creating an escalating cycle of injury and dysfunction. *Initiation Phase (direct cytotoxicity):* Systemic administration of chemotherapeutic agents inflicts direct cytotoxic damage on the highly proliferative stem and progenitor cells within the intestinal crypts due to the mechanism of chemotherapeutics targeting cell division. *Breach phase (epithelial barrier breach).* This initial insult leads to a wave of crypt cell apoptosis, resulting in a loss of intestinal regenerative capacity. The consequence is villus atrophy, crypt hypoplasia, and a physical and molecular breakdown of the epithelial barrier, characterized by the disruption of tight and adherens junction protein complexes. *Immune response phase.* The compromised barrier allows for the translocation of luminal microbial components, which, along with damage signals from dying cells, activates inflammatory pathways This triggers a positive feedback loop involving oxidative stress, lipid peroxidation, the release of pro-inflammatory cytokines, and the activation of matrix-degrading enzymes with concomitant downregulation of junction proteins that permit microbial invasion. This loop therefore drives sustained, widespread mucosal inflammation and microbial feedback. *Neuro-sensory phase (mucosal-neural crosstalk).* The inflamed and damaged mucosa releases serotonin and likely other neuroactive mediators. These molecules act on the local enteric nervous system to cause dysmotility (diarrhea, constipation) and sensitize the extrinsic primary sensory afferents in the dorsal root ganglia, leading to visceral hypersensitivity, abdominal pain, and possibly nausea, while this sensitization may also affect other peripheral tissues that are innervated by sensory afferents that also innervate the intestine. In the case of paclitaxel and anthracyclines, DRG neurons may promote recovery of the epithelium given its high regenerative potential for the intestinal epithelium when exposed to these chemotherapeutic agents (Figure 4).

Despite its profound impact on cancer treatment, the management of CIGT remains a significant, unmet clinical need. The current standard of care is largely limited to supportive measures and palliative symptom management, such as anti-emetics, anti-diarrhea medications, and analgesia. These approaches do not address the underlying pathobiology and dose reductions or treatment delays that may compromise therapeutic efficacy.

The integrated pathobiological model presented here reveals a multitude of potential targets for the development of novel, mechanism-based therapies. Interventions could be designed to act at each phase of the cascade. Strategies could focus on protecting the crypt stem cell population or inhibiting pro-apoptotic pathways specifically activated by chemotherapy in epithelial cells, by targeting the amplification loop by inhibiting key inflammatory cytokines (e.g., anti-TNF-α therapy), neutralizing ROS and preventing lipid peroxidation (antioxidants), restoring a healthy gut microbiome (probiotics, prebiotics, or fecal microbiota transplantation), or inhibiting the activity of MMPs. The symptoms of pain and dysmotility could be addressed by blocking the receptors for key neuro-sensitizing mediators, such as P2X3 receptor antagonists, therapies that target NGF or ATP, or exploring more specific serotonin receptor subtypes as therapeutic option, besides those already on the market targeting 5-HT3. The development of effective preventative and therapeutic strategies for CIGT is one of the most pressing challenges in supportive cancer care. A successful intervention would not only alleviate significant patient suffering but would also have the direct effect of enabling more patients to complete their planned chemotherapy regimens at the intended dose intensity. By preserving the efficacy of anticancer treatments, the effective management of CIGT holds the promise of improving overall cancer survival rates. Continued research into the intricate molecular and cellular interactions that drive this debilitating toxicity is therefore of paramount importance.

## References

[1] He Y Zheng J Ye B Dai Y Nie K . Chemotherapy-induced gastrointestinal toxicity: pathogenesis and current management. Biochem Pharmacology (2023) 216:115787. 10.1016/j.bcp.2023.115787 37666434

[2] Akbarali HI Muchhala KH Jessup DK Cheatham S . Chemotherapy induced gastrointestinal toxicities. Adv Cancer Res (2022) 155:131–66. 10.1016/bs.acr.2022.02.007 35779873 PMC10033220

[3] Koselke EA . Chemotherapy-induced diarrhea: options for treatment and prevention. J Hematol Oncol Pharmacol (2012) 2(4):143–51.

[4] Sanchez-Lara K Ugalde-Morales E Motola-Kuba D Green D . Gastrointestinal symptoms and weight loss in cancer patients receiving chemotherapy. Br J Nutr (2013) 109(5):894–7. 10.1017/S0007114512002073 22691288

[5] Yasuda M Kato S Yamanaka N Iimori M Matsumoto K Utsumi D 5-HT(3) receptor antagonists ameliorate 5-fluorouracil-induced intestinal mucositis by suppression of apoptosis in murine intestinal crypt cells. Br J Pharmacol (2013) 168(6):1388–400. 10.1111/bph.12019 23072534 PMC3596644

[6] Wei L Wen XS Xian CJ . Chemotherapy-induced intestinal microbiota dysbiosis impairs mucosal homeostasis by modulating toll-like receptor signaling pathways. Int J Mol Sci (2021) 22(17):9474. 10.3390/ijms22179474 34502383 PMC8431669

[7] van Vliet MJ Harmsen HJ de Bont ES Tissing WJ . The role of intestinal microbiota in the development and severity of chemotherapy-induced mucositis. Plos Pathog (2010) 6(5):e1000879. 10.1371/journal.ppat.1000879 20523891 PMC2877735

[8] Bajic JE Johnston IN Howarth GS Hutchinson MR . From the bottom-up: chemotherapy and gut-brain axis dysregulation. Front Behav Neurosci (2018) 12:104. 10.3389/fnbeh.2018.00104 29872383 PMC5972222

[9] Dahlgren D Sjoblom M Hellstrom PM Lennernas H . Chemotherapeutics-induced intestinal mucositis: pathophysiology and potential treatment strategies. Front Pharmacol (2021) 12:681417. 10.3389/fphar.2021.681417 34017262 PMC8129190

[10] Hilarius DL Kloeg PH van der Wall E van den Heuvel JJ Gundy CM Aaronson NK . Chemotherapy-induced nausea and vomiting in daily clinical practice: a community hospital-based study. Support Care Cancer (2012) 20(1):107–17. 10.1007/s00520-010-1073-9 21258948 PMC3223596

[11] Iihara H Shimokawa M Hayashi T Kawazoe H Saeki T Aiba K A nationwide, multicenter registry study of antiemesis for carboplatin-based chemotherapy-induced nausea and vomiting in Japan. Oncologist (2020) 25(2):e373–e80. 10.1634/theoncologist.2019-0292 32043774 PMC7011617

[12] Wardill HR Sonis ST Blijlevens NMA Van Sebille YZA Ciorba MA Loeffen EAH Prediction of mucositis risk secondary to cancer therapy: a systematic review of current evidence and call to action. Support Care Cancer (2020) 28(11):5059–73. 10.1007/s00520-020-05579-7 32592033

[13] Han CJ Ning X Burd CE Spakowicz DJ Tounkara F Kalady MF Chemotoxicity and associated risk factors in colorectal cancer: a systematic review and meta-analysis. Cancers (Basel) (2024) 16(14):2597. 10.3390/cancers16142597 39061235 PMC11274507

[14] Escobar Y Cajaraville G Virizuela JA Alvarez R Munoz A Olariaga O Incidence of chemotherapy-induced nausea and vomiting with moderately emetogenic chemotherapy: ADVICE (actual data of vomiting incidence by chemotherapy evaluation) study. Support Care Cancer (2015) 23(9):2833–40. 10.1007/s00520-015-2809-3 26081597 PMC4519584

[15] Patel P Robinson PD Wahib N Cheung P Wong T Cabral S Interventions for the prevention of acute phase chemotherapy-induced nausea and vomiting in adult and pediatric patients: a systematic review and meta-analysis. Support Care Cancer (2022) 30(11):8855–69. 10.1007/s00520-022-07287-w 35953731 PMC10153509

[16] Sougiannis AT VanderVeen BN Davis JM Fan D Murphy EA . Understanding chemotherapy-induced intestinal mucositis and strategies to improve gut resilience. Am J Physiol Gastrointest Liver Physiol (2021) 320(5):G712–G9. 10.1152/ajpgi.00380.2020 33471628 PMC8202195

[17] El-Rayes BF Zalupski MM Manza SG Rusin B Ferris AM Vaishampayan U Phase-II study of dose attenuated schedule of irinotecan, capecitabine, and celecoxib in advanced colorectal cancer. Cancer Chemotherapy Pharmacology (2008) 61(2):283–9. 10.1007/s00280-007-0472-1 17429629 PMC3860285

[18] Saltz LB Cox JV Blanke C Rosen LS Fehrenbacher L Moore MJ Irinotecan plus fluorouracil and leucovorin for metastatic colorectal cancer. Irinotecan study group. N Engl J Med (2000) 343(13):905–14. 10.1056/NEJM200009283431302 11006366

[19] Yang X Chen J Wang Y Wu Y Zhang J . Managing irinotecan-induced diarrhea: a comprehensive review of therapeutic interventions in cancer treatment. Pharmaceuticals (Basel) (2025) 18(3):359. 10.3390/ph18030359 40143136 PMC11944746

[20] Stein A Voigt W Jordan K . Chemotherapy-induced diarrhea: pathophysiology, frequency and guideline-based management. Ther Adv Med Oncol (2010) 2(1):51–63. 10.1177/1758834009355164 21789126 PMC3126005

[21] Ontario CC . Irinotecan. (2026)

[22] Hoff PM Ansari R Batist G Cox J Kocha W Kuperminc M Comparison of oral capecitabine *versus* intravenous fluorouracil plus leucovorin as first-line treatment in 605 patients with metastatic colorectal cancer: results of a randomized phase III study. J Clin Oncol (2001) 19(8):2282–92. 10.1200/JCO.2001.19.8.2282 11304782

[23] Olver I Paska W Depierre A Seitz JF Stewart DJ Goedhals L A multicentre, double-blind study comparing placebo, ondansetron and ondansetron plus dexamethasone for the control of cisplatin-induced delayed emesis. Ondansetron delayed emesis study group. Ann Oncol (1996) 7(9):945–52. 10.1093/oxfordjournals.annonc.a010798 9006746

[24] Gandara DR Harvey WH Monaghan GG Perez EA Hesketh PJ . Delayed emesis following high-dose cisplatin: a double-blind randomised comparative trial of ondansetron (GR 38032F) *versus* placebo. Eur J Cancer (1993) 29A(Suppl. 1):S35–8. 10.1016/s0959-8049(05)80259-x 8427724

[25] Hari G Prakash SKD Kiran PK Arun V Yadav D Gopi A . Asian Pacific journal of cancer care. (2024) 9(1).

[26] Sommariva S Pongiglione B Tarricone R . Impact of chemotherapy-induced nausea and vomiting on health-related quality of life and resource utilization: a systematic review. Crit Rev Oncol Hematol (2016) 99:13–36. 10.1016/j.critrevonc.2015.12.001 26697988

[27] Keefe DM Elting LS Nguyen HT Grunberg SM Aprile G Bonaventura A Risk and outcomes of chemotherapy-induced diarrhea (CID) among patients with colorectal cancer receiving multi-cycle chemotherapy. Cancer Chemotherapy Pharmacology (2014) 74(4):675–80. 10.1007/s00280-014-2526-5 25055935

[28] Zeien J Qiu W Triay M Dhaibar HA Cruz-Topete D Cornett EM Clinical implications of chemotherapeutic agent organ toxicity on perioperative care. Biomed Pharmacother (2022) 146:112503. 10.1016/j.biopha.2021.112503 34922113 PMC11118057

[29] Wonders KY Schmitz K Harness J . Dose delays, dose reductions, and relative total dose intensity in patients with advanced cancer who exercised during neoadjuvant chemotherapy treatment. Integr Cancer Ther (2023) 22:15347354231168368. 10.1177/15347354231168368 37077136 PMC10126785

[30] Lyman GH . Impact of chemotherapy dose intensity on cancer patient outcomes. J Natl Compr Canc Netw (2009) 7(1):99–108. 10.6004/jnccn.2009.0009 19176210

[31] Nielson CM Bylsma LC Fryzek JP Saad HA Crawford J . Relative dose intensity of chemotherapy and survival in patients with advanced stage solid tumor cancer: a systematic review and meta-analysis. Oncologist (2021) 26(9):e1609–e18. 10.1002/onco.13822 33973301 PMC8417866

[32] Havrilesky LJ Reiner M Morrow PK Watson H Crawford J . A review of relative dose intensity and survival in patients with metastatic solid tumors. Crit Rev Oncol Hematol (2015) 93(3):203–10. 10.1016/j.critrevonc.2014.10.006 25459671

[33] Crawford J Denduluri N Patt D Jiao X Morrow PK Garcia J Relative dose intensity of first-line chemotherapy and overall survival in patients with advanced non-small-cell lung cancer. Support Care Cancer (2020) 28(2):925–32. 10.1007/s00520-019-04875-1 31172284 PMC6954126

[34] Bonadonna G Valagussa P Moliterni A Zambetti M Brambilla C . Adjuvant cyclophosphamide, methotrexate, and fluorouracil in node-positive breast cancer: the results of 20 years of follow-up. N Engl J Med (1995) 332(14):901–6. 10.1056/NEJM199504063321401 7877646

[35] Sonis ST . The pathobiology of mucositis. Nat Rev Cancer (2004) 4(4):277–84. 10.1038/nrc1318 15057287

[36] Singh P Nayernama A Christopher Jones S Amiri Kordestani L Fedenko K Prowell T Fatal neutropenic enterocolitis associated with docetaxel use: a review of cases reported to the United States food and drug administration adverse event reporting system. J Oncol Pharm Pract (2020) 26(4):923–8. 10.1177/1078155219879494 31594460

[37] Pugliese N Salvatore P Iula DV Catania MR Chiurazzi F Della Pepa R Ultrasonography-driven combination antibiotic therapy with tigecycline significantly increases survival among patients with neutropenic enterocolitis following cytarabine-containing chemotherapy for the remission induction of acute myeloid leukemia. Cancer Med (2017) 6(7):1500–11. 10.1002/cam4.1063 28556623 PMC5504336

[38] Hanna TP King WD Thibodeau S Jalink M Paulin GA Harvey-Jones E Mortality due to cancer treatment delay: systematic review and meta-analysis. BMJ (2020) 371:m4087. 10.1136/bmj.m4087 33148535 PMC7610021

[39] van der Flier LG Clevers H . Stem cells, self-renewal, and differentiation in the intestinal epithelium. Annu Rev Physiol (2009) 71:241–60. 10.1146/annurev.physiol.010908.163145 18808327

[40] Keefe DM Gibson RJ Hauer-Jensen M . Gastrointestinal mucositis. Semin Oncol Nurs (2004) 20(1):38–47. 10.1053/j.soncn.2003.10.007 15038516

[41] Ijiri K Potten CS . Further studies on the response of intestinal crypt cells of different hierarchical status to eighteen different cytotoxic agents. Br J Cancer (1987) 55(2):113–23. 10.1038/bjc.1987.25 3814484 PMC2002086

[42] Yoneda J Nishikawa S Kurihara S . Oral administration of cystine and theanine attenuates 5-fluorouracil-induced intestinal mucositis and diarrhea by suppressing both glutathione level decrease and ROS production in the small intestine of mucositis mouse model. BMC Cancer (2021) 21(1):1343. 10.1186/s12885-021-09057-z 34922485 PMC8684148

[43] Long BH Fairchild CR . Paclitaxel inhibits progression of mitotic cells to G1 phase by interference with spindle formation without affecting other microtubule functions during anaphase and telephase. Cancer Research (1994) 54(16):4355–61. 7913875

[44] Chen JG Horwitz SB . Differential mitotic responses to microtubule-stabilizing and -destabilizing drugs. Cancer Research (2002) 62(7):1935–8. 11929805

[45] Hornick JE Bader JR Tribble EK Trimble K Breunig JS Halpin ES Live-cell analysis of mitotic spindle formation in taxol-treated cells. Cell Motil Cytoskeleton (2008) 65(8):595–613. 10.1002/cm.20283 18481305 PMC2753270

[46] Jordan MA Wendell K Gardiner S Derry WB Copp H Wilson L . Mitotic block induced in HeLa cells by low concentrations of paclitaxel (taxol) results in abnormal mitotic exit and apoptotic cell death. Cancer Research (1996) 56(4):816–25. 8631019

[47] Varbiro G Veres B Gallyas F Sumegi B . Direct effect of taxol on free radical formation and mitochondrial permeability transition. Free Radic Biol Med (2001) 31(4):548–58. 10.1016/s0891-5849(01)00616-5 11498288

[48] André N Carré M Brasseur G Pourroy B Kovacic H Briand C Paclitaxel targets mitochondria upstream of caspase activation in intact human neuroblastoma cells. FEBS Letters (2002) 532(1-2):256–60. 10.1016/s0014-5793(02)03691-8 12459501

[49] Jordan MA Wilson L . Microtubules as a target for anticancer drugs. Nat Rev Cancer (2004) 4(4):253–65. 10.1038/nrc1317 15057285

[50] Weaver BA . How taxol/paclitaxel kills cancer cells. Mol Biol Cell (2014) 25(18):2677–81. 10.1091/mbc.E14-04-0916 25213191 PMC4161504

[51] Daniels JA Gibson MK Xu L Sun S Canto MI Heath E Gastrointestinal tract epithelial changes associated with taxanes: marker of drug toxicity *versus* effect. Am J Surg Pathol (2008) 32(3):473–7. 10.1097/PAS.0b013e3181582331 18300801

[52] Mason KA Milas L Peters LJ . Effect of paclitaxel (Taxol) alone and in combination with radiation on the gastrointestinal mucosa. Int J Radiat Oncol Biol Phys (1995) 32(5):1381–9. 10.1016/0360-3016(95)00037-Y 7635778

[53] Levin G Shirvan A Grimberg H Reshef A Yogev-Falach M Cohen A Novel fluorescence molecular imaging of chemotherapy-induced intestinal apoptosis. J Biomed Opt (2009) 14(5):054019. 10.1117/1.3253303 19895121

[54] Yu WD Lee S Cho HS Kwon O Lim JH Jung CR Drug-induced gastrointestinal toxicity and barrier integrity: cytoskeleton-mediated impairment in a clinically relevant human intestinal epithelium model. Exp Mol Med (2026) 58(2):487–500. 10.1038/s12276-025-01635-6 41680471 PMC12992830

[55] Lee HY Lee YH Kim MJ Kim HK . Secondary prophylaxis of docetaxel induced diarrhea with loperamide: case report. J Korean Med Sci (2013) 28(10):1549–51. 10.3346/jkms.2013.28.10.1549 24133365 PMC3792614

[56] Yamazawa K Kanno H Seki K Kuzuta T Matsui H Sekiya S . Life-threatening clostridium difficile-associated diarrhea induced by paclitaxel-carboplatin combination chemotherapy. Acta Obstet Gynecol Scand (2001) 80(8):768–9. 11531624

[57] Owellen RJ Hartke CA Dickerson RM Hains FO . Inhibition of tubulin-microtubule polymerization by drugs of the vinca alkaloid class. Cancer Research (1976) 36(4):1499–502. 1260766

[58] Playford RJ Marchbank T Mandir N Higham A Meeran K Ghatei MA Effects of keratinocyte growth factor (KGF) on gut growth and repair. J Pathol (1998) 184(3):316–22. 10.1002/(SICI)1096-9896(199803)184:3<316::AID-PATH3>3.0.CO;2-# 9614385

[59] Harmon BV Takano YS Winterford CM Potten CS . Cell death induced by vincristine in the intestinal crypts of mice and in a human Burkitt's lymphoma cell line. Cell Prolif (1992) 25(6):523–36. 10.1111/j.1365-2184.1992.tb01457.x 1457603

[60] Selimovic D Hassan M Haikel Y Hengge UR . Taxol-induced mitochondrial stress in melanoma cells is mediated by activation of c-Jun N-terminal kinase (JNK) and p38 pathways *via* uncoupling protein 2. Cell Signal (2008) 20(2):311–22. 10.1016/j.cellsig.2007.10.015 18068334

[61] Naldoni C Guidotti I Matteoli M Di Bello D Scatolini V Esposti V Induction of genotoxic damage and ROS production in HCT116 TP53+/+ and HCT116TP53-/- colorectal cancer cell lines by anticancer drugs. Mutagenesis (2026). 10.1093/mutage/geag014 41870588

[62] Chen X Wu Y Dong H Zhang CY Zhang Y . Platinum-based agents for individualized cancer treatment. Curr Mol Med (2013) 13(10):1603–12. 10.2174/1566524013666131111125515 24206132

[63] Wang D Lippard SJ . Cellular processing of platinum anticancer drugs. Nat Rev Drug Discov (2005) 4(4):307–20. 10.1038/nrd1691 15789122

[64] Millard JT Wilkes EE . cis- and trans-diamminedichloroplatinum(II) interstrand cross-linking of a defined sequence nucleosomal core particle. Biochemistry (2000) 39(51):16046–55. 10.1021/bi0022285 11123932

[65] Kasparkova J Fojta M Farrell N Brabec V . Differential recognition by the tumor suppressor protein p53 of DNA modified by the novel antitumor trinuclear platinum drug BBR3464 and cisplatin. Nucleic Acids Res (2004) 32(18):5546–52. 10.1093/nar/gkh896 15486204 PMC524304

[66] Levine AJ . p53: 800 million years of evolution and 40 years of discovery. Nat Rev Cancer (2020) 20(8):471–80. 10.1038/s41568-020-0262-1 32404993

[67] da Costa A Chowdhury D Shapiro GI D'Andrea AD Konstantinopoulos PA . Targeting replication stress in cancer therapy. Nat Rev Drug Discov (2023) 22(1):38–58. 10.1038/s41573-022-00558-5 36202931 PMC11132912

[68] Meng X Gao JZ Gomendoza SMT Li JW Yang S . Recent advances of WEE1 inhibitors and statins in cancers with p53 mutations. Front Med (Lausanne) (2021) 8:737951. 10.3389/fmed.2021.737951 34671620 PMC8520942

[69] Keefe DM Brealey J Goland GJ Cummins AG . Chemotherapy for cancer causes apoptosis that precedes hypoplasia in crypts of the small intestine in humans. Gut (2000) 47(5):632–7. 10.1136/gut.47.5.632 11034578 PMC1728102

[70] Oronsky B Caroen S Oronsky A Dobalian VE Oronsky N Lybeck M Electrolyte disorders with platinum-based chemotherapy: mechanisms, manifestations and management. Cancer Chemotherapy Pharmacology (2017) 80(5):895–907. 10.1007/s00280-017-3392-8 28730291 PMC5676816

[71] Shahid F Farooqui Z Khan F . Cisplatin-induced gastrointestinal toxicity: an update on possible mechanisms and on available gastroprotective strategies. Eur Journal Pharmacology (2018) 827:49–57. 10.1016/j.ejphar.2018.03.009 29530589

[72] McKeage MJ . Comparative adverse effect profiles of platinum drugs. Drug Saf (1995) 13(4):228–44. 10.2165/00002018-199513040-00003 8573296

[73] Abu-Sbeih H Mallepally N Goldstein R Chen E Tang T Dike UK Gastrointestinal toxic effects in patients with cancer receiving platinum-based therapy. J Cancer (2020) 11(11):3144–50. 10.7150/jca.37777 32231718 PMC7097936

[74] Yanez JA Teng XW Roupe KA Fariss MW Davies NM . Chemotherapy induced gastrointestinal toxicity in rats: involvement of mitochondrial DNA, gastrointestinal permeability and cyclooxygenase -2. J Pharm Pharm Sci (2003) 6(3):308–14. 14738710

[75] Airoldi M Barone G Gennaro G Giuliani AM Giustini M . Interaction of doxorubicin with polynucleotides. A spectroscopic study. Biochemistry (2014) 53(13):2197–207. 10.1021/bi401687v 24641674

[76] Goodman MF Lee GM . Adriamycin interactions with T4 DNA polymerase. Two modes of template-mediated inhibition. The J Biological Chemistry (1977) 252(8):2670–4. 323251

[77] Tarr M van Helden PD . Inhibition of transcription by adriamycin is a consequence of the loss of negative superhelicity in DNA mediated by topoisomerase II. Mol Cellular Biochemistry (1990) 93(2):141–6. 10.1007/BF00226185 2161074

[78] Zunino F Capranico G . DNA topoisomerase II as the primary target of anti-tumor anthracyclines. Anticancer Drug Des (1990) 5(4):307–17. 1963303

[79] Tewey KM Rowe TC Yang L Halligan BD Liu LF . Adriamycin-induced DNA damage mediated by Mammalian DNA topoisomerase II. Science (1984) 226(4673):466–8. 10.1126/science.6093249 6093249

[80] Davies KJ Doroshow JH . Redox cycling of anthracyclines by cardiac mitochondria. I. Anthracycline radical formation by NADH dehydrogenase. The J Biological Chemistry (1986) 261(7):3060–7. 3456345

[81] Berlin V Haseltine WA . Reduction of adriamycin to a semiquinone-free radical by NADPH cytochrome P-450 reductase produces DNA cleavage in a reaction mediated by molecular oxygen. The J Biological Chemistry (1981) 256(10):4747–56. 6262301

[82] Dekaney CM Gulati AS Garrison AP Helmrath MA Henning SJ . Regeneration of intestinal stem/progenitor cells following doxorubicin treatment of mice. Am J Physiol Gastrointest Liver Physiol (2009) 297(3):G461–70. 10.1152/ajpgi.90446.2008 19589945 PMC2739827

[83] Alzahrani SM Al Doghaither HA Al-Ghafari AB Pushparaj PN . 5-Fluorouracil and capecitabine therapies for the treatment of colorectal cancer. Oncol Rep (2023) 50(4). 10.3892/or.2023.8612 37594133

[84] Iacovelli R Pietrantonio F Palazzo A Maggi C Ricchini F de Braud F Incidence and relative risk of grade 3 and 4 diarrhoea in patients treated with capecitabine or 5-fluorouracil: a meta-analysis of published trials. Br J Clin Pharmacol (2014) 78(6):1228–37. 10.1111/bcp.12449 24962653 PMC4256612

[85] Sakai H Sagara A Matsumoto K Hasegawa S Sato K Nishizaki M 5-Fluorouracil induces diarrhea with changes in the expression of inflammatory cytokines and aquaporins in mouse intestines. PLoS One (2013) 8(1):e54788. 10.1371/journal.pone.0054788 23382968 PMC3559799

[86] Andreyev J Ross P Donnellan C Lennan E Leonard P Waters C Guidance on the management of diarrhoea during cancer chemotherapy. Lancet Oncol (2014) 15(10):e447–60. 10.1016/S1470-2045(14)70006-3 25186048

[87] Chen M Li Y Chen P . Restore intestinal steady-state: new advances in the clinical management of chemotherapy-associated diarrhea and constipation. J Mol Histol (2025) 56(2):101. 10.1007/s10735-025-10367-w 40056250 PMC11890403

[88] Gibson RJ Bowen JM Inglis MR Cummins AG Keefe DM . Irinotecan causes severe small intestinal damage, as well as colonic damage, in the rat with implanted breast cancer. J Gastroenterol Hepatol (2003) 18(9):1095–100. 10.1046/j.1440-1746.2003.03136.x 12911669

[89] Wardill HR Bowen JM Al-Dasooqi N Sultani M Bateman E Stansborough R Irinotecan disrupts tight junction proteins within the gut: implications for chemotherapy-induced gut toxicity. Cancer Biol Ther (2014) 15(2):236–44. 10.4161/cbt.27222 24316664 PMC3928140

[90] Arifa RD Madeira MF de Paula TP Lima RL Tavares LD Menezes-Garcia Z Inflammasome activation is reactive oxygen species dependent and mediates irinotecan-induced mucositis through IL-1beta and IL-18 in mice. The Am Journal Pathology (2014) 184(7):2023–34. 10.1016/j.ajpath.2014.03.012 24952429

[91] Hsieh CJ Cirrincione AM Vlach MR Diaz AC Schmidt NA Li X Paclitaxel neurotoxicity is triggered by epidermal EG5-dependent microtubule fasciculation and X-ROS formation. Res Sq (2025). V1 (one). 10.21203/rs.3.rs-5470731/v1 40894047 PMC12393485

[92] Lisse TS Middleton LJ Pellegrini AD Martin PB Spaulding EL Lopes O Paclitaxel-induced epithelial damage and ectopic MMP-13 expression promotes neurotoxicity in zebrafish. Proc Natl Acad Sci USA (2016) 113(15):E2189–98. 10.1073/pnas.1525096113 27035978 PMC4839466

[93] Mikesell AR Isaeva E Schulte ML Menzel AD Sriram A Prahl MM Increased keratinocyte activity and PIEZO1 signaling contribute to paclitaxel-induced mechanical hypersensitivity. Sci Transl Med (2024) 16(777):eadn5629. 10.1126/scitranslmed.adn5629 39661703

[94] Bodiga VL Bodiga S Surampudi S Boindala S Putcha U Nagalla B Effect of vitamin supplementation on cisplatin-induced intestinal epithelial cell apoptosis in Wistar/NIN rats. Nutrition (2012) 28(5):572–80. 10.1016/j.nut.2011.09.007 22189195

[95] Hilkens PH ven den Bent MJ . Chemotherapy-induced peripheral neuropathy. J Peripher Nerv Syst (1997) 2(4):350–61. 10975744

[96] Gornstein E Schwarz TL . The paradox of paclitaxel neurotoxicity: mechanisms and unanswered questions. Neuropharmacology (2014) 76(Pt A):175–83. 10.1016/j.neuropharm.2013.08.016 23978385

[97] Staff NP Fehrenbacher JC Caillaud M Damaj MI Segal RA Rieger S . Pathogenesis of paclitaxel-induced peripheral neuropathy: a current review of *in vitro* and *in vivo* findings using rodent and human model systems. Exp Neurology (2020) 324:113121. 10.1016/j.expneurol.2019.113121 31758983 PMC6993945

[98] Khiati S Dalla Rosa I Sourbier C Ma X Rao VA Neckers LM Mitochondrial topoisomerase I (top1mt) is a novel limiting factor of doxorubicin cardiotoxicity. Clin Cancer Res (2014) 20(18):4873–81. 10.1158/1078-0432.CCR-13-3373 24714774 PMC4167185

[99] Sarraf CE Ansari TW Conway P Notay M Hill S Alison MR . Bromodeoxyuridine-labelled apoptosis after treatment with antimetabolites in two murine tumours and in small intestinal crypts. Br J Cancer (1993) 68(4):678–80. 10.1038/bjc.1993.408 8398692 PMC1968621

[100] Bhat AH Dar KB Anees S Zargar MA Masood A Sofi MA Oxidative stress, mitochondrial dysfunction and neurodegenerative diseases; a mechanistic insight. Biomed Pharmacother (2015) 74:101–10. 10.1016/j.biopha.2015.07.025 26349970

[101] Ullman TA Itzkowitz SH . Intestinal inflammation and cancer. Gastroenterology (2011) 140(6):1807–16. 10.1053/j.gastro.2011.01.057 21530747

[102] Logan RM Gibson RJ Bowen JM Stringer AM Sonis ST Keefe DM . Characterisation of mucosal changes in the alimentary tract following administration of irinotecan: implications for the pathobiology of mucositis. Cancer Chemotherapy Pharmacology (2008) 62(1):33–41. 10.1007/s00280-007-0570-0 17703303

[103] Vijayalakshmi B Sesikeran B Udaykumar P Kalyanasundaram S Raghunath M . Chronic low vitamin intake potentiates cisplatin-induced intestinal epithelial cell apoptosis in WNIN rats. World Journal Gastroenterology : WJG (2006) 12(7):1078–85. 10.3748/wjg.v12.i7.1078 16534849 PMC4087900

[104] Cray P Sheahan BJ Cortes JE Dekaney CM . Doxorubicin increases permeability of murine small intestinal epithelium and cultured T84 monolayers. Sci Rep (2020) 10(1):21486. 10.1038/s41598-020-78473-1 33293626 PMC7722747

[105] Li HL Lu L Wang XS Qin LY Wang P Qiu SP Alteration of gut microbiota and inflammatory cytokine/chemokine profiles in 5-Fluorouracil induced intestinal mucositis. Front Cell Infect Microbiol (2017) 7:455. 10.3389/fcimb.2017.00455 29124041 PMC5662589

[106] Prisciandaro LD Geier MS Chua AE Butler RN Cummins AG Sander GR Probiotic factors partially prevent changes to caspases 3 and 7 activation and transepithelial electrical resistance in a model of 5-fluorouracil-induced epithelial cell damage. Support Care Cancer (2012) 20(12):3205–10. 10.1007/s00520-012-1446-3 22526145

[107] Soares PM Mota JM Souza EP Justino PF Franco AX Cunha FQ Inflammatory intestinal damage induced by 5-fluorouracil requires IL-4. Cytokine (2013) 61(1):46–9. 10.1016/j.cyto.2012.10.003 23107827

[108] Dan Dunn J Alvarez LA Zhang X Soldati T . Reactive oxygen species and mitochondria: a nexus of cellular homeostasis. Redox Biol (2015) 6:472–85. 10.1016/j.redox.2015.09.005 26432659 PMC4596921

[109] Liu T Zhang L Joo D Sun SC . NF-kappaB signaling in inflammation. Signal Transduct Target Ther (2017) 2:17023. 10.1038/sigtrans.2017.23 29158945 PMC5661633

[110] Sultani M Stringer AM Bowen JM Gibson RJ . Anti-inflammatory cytokines: important immunoregulatory factors contributing to chemotherapy-induced gastrointestinal mucositis. Chemother Res Pract (2012) 2012:490804. 10.1155/2012/490804 22973511 PMC3437608

[111] Machado GF Pereira QC Fagundes FL Emilio-Silva MT Rodrigues VP Patino MA The anthocyanidins malvidin and cyanidin alleviate irinotecan-triggered intestinal mucositis by modulating oxidative stress and cytokine release. Int J Mol Sci (2025) 26(21):10747. 10.3390/ijms262110747 41226784 PMC12609383

[112] Shen SR Chen WJ Chu HF Wu SH Wang YR Shen TL . Amelioration of 5-fluorouracil-induced intestinal mucositis by streptococcus thermophilus ST4 in a mouse model. PLoS One (2021) 16(7):e0253540. 10.1371/journal.pone.0253540 34310611 PMC8312939

[113] Luo J Qian A Oetjen LK Yu W Yang P Feng J TRPV4 channel signaling in macrophages promotes gastrointestinal motility *via* direct effects on smooth muscle cells. Immunity (2018) 49(1):107–19 e4. 10.1016/j.immuni.2018.04.021 29958798 PMC6051912

[114] Focaccetti C Bruno A Magnani E Bartolini D Principi E Dallaglio K Effects of 5-fluorouracil on morphology, cell cycle, proliferation, apoptosis, autophagy and ROS production in endothelial cells and cardiomyocytes. PLoS One (2015) 10(2):e0115686. 10.1371/journal.pone.0115686 25671635 PMC4324934

[115] Zhang JN Li KD Cao ZJ Xu LY Zhao XL Tang F Mechanisms of magnoliae officinalis cortex volatile oil in alleviating 5-Fluorouracil-Induced mucositis *via* multi-omics approaches. Drug Des Devel Ther (2025) 19:7503–25. 10.2147/DDDT.S515605 40904544 PMC12404258

[116] Logan RM Stringer AM Bowen JM Gibson RJ Sonis ST Keefe DM . Serum levels of NFkappaB and pro-inflammatory cytokines following administration of mucotoxic drugs. Cancer Biol Ther (2008) 7(7):1139–45. 10.4161/cbt.7.7.6207 18535404

[117] Al-Sadi RM Ma TY . IL-1beta causes an increase in intestinal epithelial tight junction permeability. J Immunol (2007) 178(7):4641–9. 10.4049/jimmunol.178.7.4641 17372023 PMC3724221

[118] Visse R Nagase H . Matrix metalloproteinases and tissue inhibitors of metalloproteinases: structure, function, and biochemistry. Circ Research (2003) 92(8):827–39. 10.1161/01.RES.0000070112.80711.3D 12730128

[119] Cheon H Yu SJ Yoo DH Chae IJ Song GG Sohn J . Increased expression of pro-inflammatory cytokines and metalloproteinase-1 by TGF-beta1 in synovial fibroblasts from rheumatoid arthritis and normal individuals. Clin Experimental Immunology (2002) 127(3):547–52. 10.1046/j.1365-2249.2002.01785.x 11966774 PMC1906321

[120] Nyormoi O Mills L Bar-Eli M . An MMP-2/MMP-9 inhibitor, 5a, enhances apoptosis induced by ligands of the TNF receptor superfamily in cancer cells. Cell Death Differentiation (2003) 10(5):558–69. 10.1038/sj.cdd.4401209 12728254

[121] Staff Nph SC Dasari S Capobianco E Rieger S . Skin extracellular matrix breakdown following paclitaxel therapy in patients with chemotherapy-induced peripheral neuropathy. MDPI Cancers (2023) 15(16). 10.3390/cancers15164191 PMC10453667 37627219

[122] Al-Dasooqi N Gibson RJ Bowen JM Logan RM Stringer AM Keefe DM . Matrix metalloproteinases are possible mediators for the development of alimentary tract mucositis in the dark agouti rat. Exp Biol Med (Maywood) (2010) 235(10):1244–56. 10.1258/ebm.2010.010082 20682600

[123] Al-Azri AR Gibson RJ Bowen JM Stringer AM Keefe DM Logan RM . Involvement of matrix metalloproteinases (MMP-3 and MMP-9) in the pathogenesis of irinotecan-induced oral mucositis. J Oral Pathology and Medicine (2015) 44(6):459–67. 10.1111/jop.12255 25213123

[124] Vandenbroucke RE Dejonckheere E Van Hauwermeiren F Lodens S De Rycke R Van Wonterghem E Matrix metalloproteinase 13 modulates intestinal epithelial barrier integrity in inflammatory diseases by activating TNF. EMBO Mol Med (2013) 5(7):932–48. 10.1002/emmm.201202100 23723167 PMC3721470

[125] Becker-Pauly C Rose-John S . TNFalpha cleavage beyond TACE/ADAM17: matrix metalloproteinase 13 is a potential therapeutic target in sepsis and colitis. EMBO Mol Med (2013) 5(7):970–2. 10.1002/emmm.201302899 23757215 PMC3721467

[126] Mummidi S Das NA Carpenter AJ Yoshida T Yariswamy M Mostany R RECK suppresses interleukin-17/TRAF3IP2-mediated MMP-13 activation and human aortic smooth muscle cell migration and proliferation. J Cellular Physiology (2019) 234(12):22242–59. 10.1002/jcp.28792 31074012 PMC7276214

[127] Shindle MK Chen CC Robertson C DiTullio AE Paulus MC Clinton CM Full-thickness supraspinatus tears are associated with more synovial inflammation and tissue degeneration than partial-thickness tears. J Shoulder Elbow Surg (2011) 20(6):917–27. 10.1016/j.jse.2011.02.015 21612944 PMC3156316

[128] Uchinami H Seki E Brenner DA D'Armiento J . Loss of MMP 13 attenuates murine hepatic injury and fibrosis during cholestasis. Hepatology (2006) 44(2):420–9. 10.1002/hep.21268 16871591

[129] Montassier E Gastinne T Vangay P Al-Ghalith GA Bruley des Varannes S Massart S Chemotherapy-driven dysbiosis in the intestinal microbiome. Aliment Pharmacol Ther (2015) 42(5):515–28. 10.1111/apt.13302 26147207

[130] Wardill HR Gibson RJ Van Sebille YZ Secombe KR Coller JK White IA Irinotecan-induced gastrointestinal dysfunction and pain are mediated by common TLR4-Dependent mechanisms. Mol Cancer Ther (2016) 15(6):1376–86. 10.1158/1535-7163.MCT-15-0990 27197307

[131] Wong DV Lima-Junior RC Carvalho CB Borges VF Wanderley CW Bem AX The adaptor protein Myd88 is a key signaling molecule in the pathogenesis of irinotecan-induced intestinal mucositis. PLoS One (2015) 10(10):e0139985. 10.1371/journal.pone.0139985 26440613 PMC4595146

[132] Chang CT Ho TY Lin H Liang JA Huang HC Li CC 5-Fluorouracil induced intestinal mucositis *via* nuclear factor-kappaB activation by transcriptomic analysis and *in vivo* bioluminescence imaging. PLoS One (2012) 7(3):e31808. 10.1371/journal.pone.0031808 22412841 PMC3296709

[133] Cryan JF O'Riordan KJ Cowan CSM Sandhu KV Bastiaanssen TFS Boehme M The microbiota-gut-brain axis. Physiol Reviews (2019) 99(4):1877–2013. 10.1152/physrev.00018.2018 31460832

[134] Filetti M Lombardi P Giusti R Falcone R Scotte F Giannarelli D Efficacy and safety of antiemetic regimens for highly emetogenic chemotherapy-induced nausea and vomiting: a systematic review and network meta-analysis. Cancer Treat Rev (2023) 115:102512. 10.1016/j.ctrv.2023.102512 36774658

[135] McQuade RM Al Thaalibi M Nurgali K . Impact of chemotherapy-induced enteric nervous system toxicity on gastrointestinal mucositis. Curr Opin Support Palliat Care (2020) 14(3):293–300. 10.1097/SPC.0000000000000515 32769620

[136] Rahman AA Masango P Stavely R Bertrand P Page A Nurgali K . Oxaliplatin-induced damage to the gastric innervation: role in nausea and vomiting. Biomolecules (2023) 13(2):276. 10.3390/biom13020276 36830645 PMC9952961

[137] Wafai L Taher M Jovanovska V Bornstein JC Dass CR Nurgali K . Effects of oxaliplatin on mouse myenteric neurons and colonic motility. Front Neurosci (2013) 7:30. 10.3389/fnins.2013.00030 23486839 PMC3594784

[138] Yang D Jacobson A Meerschaert KA Sifakis JJ Wu M Chen X Nociceptor neurons direct goblet cells *via* a CGRP-RAMP1 axis to drive mucus production and gut barrier protection. Cell (2022) 185(22):4190–205 e25. 10.1016/j.cell.2022.09.024 36243004 PMC9617795

[139] Li Y Marri T North RY Rhodes HR Uhelski ML Tatsui CE Chemotherapy-induced peripheral neuropathy in a dish: dorsal root ganglion cells treated *in vitro* with paclitaxel show biochemical and physiological responses parallel to that seen *in vivo* . Pain (2021) 162(1):84–96. 10.1097/j.pain.0000000000002005 32694383 PMC7744394

[140] Stojanovska V McQuade RM Miller S Nurgali K . Effects of oxaliplatin treatment on the myenteric plexus innervation and glia in the murine distal Colon. J Histochem Cytochem (2018) 66(10):723–36. 10.1369/0022155418774755 29741434 PMC6158630

[141] Rossato MF Rigo FK Oliveira SM Guerra GP Silva CR Cunha TM Participation of transient receptor potential vanilloid 1 in paclitaxel-induced acute visceral and peripheral nociception in rodents. Eur Journal Pharmacology (2018) 828:42–51. 10.1016/j.ejphar.2018.03.033 29577893

[142] Vera G Nurgali K Abalo R . Chemotherapy-induced neuropathy affecting the gastrointestinal tract. Neurogastroenterol Motil (2025) 37(8):e14976. 10.1111/nmo.14976 39651634 PMC12287903

[143] Lin YM Fu Y Wu CC Xu GY Huang LY Shi XZ . Colon distention induces persistent visceral hypersensitivity by mechanotranscription of pain mediators in colonic smooth muscle cells. Am J Physiol Gastrointest Liver Physiol (2015) 308(5):G434–41. 10.1152/ajpgi.00328.2014 25540231 PMC4346753

[144] Song Y Fothergill LJ Lee KS Liu BY Koo A Perelis M Stratification of enterochromaffin cells by single-cell expression analysis. Elife (2025) 12. 10.7554/eLife.90596 40184163 PMC11970908

[145] Bearcroft CP Domizio P Mourad FH Andre EA Farthing MJ . Cisplatin impairs fluid and electrolyte absorption in rat small intestine: a role for 5-hydroxytryptamine. Gut (1999) 44(2):174–9. 10.1136/gut.44.2.174 9895375 PMC1727387

[146] Obara Y Machida T Takano Y Shiga S Suzuki A Hamaue N Cisplatin increases the number of enterochromaffin cells containing substance P in rat intestine. Naunyn Schmiedebergs Arch Pharmacol (2018) 391(8):847–58. 10.1007/s00210-018-1493-5 29766222

[147] Browning KN . Role of central vagal 5-HT3 receptors in gastrointestinal physiology and pathophysiology. Front Neurosci (2015) 9:413. 10.3389/fnins.2015.00413 26578870 PMC4625078

[148] Caliskaner ZO . Evaluation of emetogenic mechanisms of antineoplastic drugs on 5-HT3A receptor using a structure-based computational approach. J Clin Pract Res (2025) 47(3):262–71. 10.14744/cpr.2025.49099 41256073 PMC12478664

[149] Zeitz KP Guy N Malmberg AB Dirajlal S Martin WJ Sun L The 5-HT3 subtype of serotonin receptor contributes to nociceptive processing *via* a novel subset of myelinated and unmyelinated nociceptors. J Neurosci (2002) 22(3):1010–9. 10.1523/JNEUROSCI.22-03-01010.2002 11826129 PMC6758503

[150] Hashimoto Y Maeda K Shimomura O Miyazaki Y Hashimoto S Oda T Development of the serotonin release assay with enterochromaffin cell-rich human intestinal organoids for the risk evaluation of drug-induced emesis. Toxicol Sci (2025) 206(2):299–312. 10.1093/toxsci/kfae158 39689026

[151] Horn CC Richardson EJ Andrews PL Friedman MI . Differential effects on gastrointestinal and hepatic vagal afferent fibers in the rat by the anti-cancer agent cisplatin. Auton Neurosci (2004) 115(1-2):74–81. 10.1016/j.autneu.2004.08.011 15507408

[152] Williams EK Chang RB Strochlic DE Umans BD Lowell BB Liberles SD . Sensory neurons that detect stretch and nutrients in the digestive system. Cell (2016) 166(1):209–21. 10.1016/j.cell.2016.05.011 27238020 PMC4930427

[153] Wolfson RL Abdelaziz A Rankin G Kushner S Qi L Mazor O DRG afferents that mediate physiologic and pathologic mechanosensation from the distal Colon. Cell (2023) 186(16):3368–85 e18. 10.1016/j.cell.2023.07.007 37541195 PMC10440726

[154] Cavaletti G Cavalletti E Oggioni N Sottani C Minoia C D'Incalci M Distribution of paclitaxel within the nervous system of the rat after repeated intravenous administration. Neurotoxicology (2000) 21(3):389–93. 10894128

[155] Zhang H Li Y de Carvalho-Barbosa M Kavelaars A Heijnen CJ Albrecht PJ Dorsal root ganglion infiltration by macrophages contributes to paclitaxel chemotherapy-induced peripheral neuropathy. J Pain (2016) 17(7):775–86. 10.1016/j.jpain.2016.02.011 26979998 PMC4939513

[156] Patai R Kiss T Gulej R Nyul-Toth A Csik B Chandragiri SS Transcriptomic profiling of senescence effects on blood-brain barrier-related gene expression in brain capillary endothelial cells in a mouse model of paclitaxel-induced chemobrain. Geroscience (2025) 47(3):3677–91. 10.1007/s11357-025-01561-5 39976844 PMC12181502

[157] Patai R Csik B Nyul-Toth A Gulej R Vali Kordestan K Chandragiri SS Persisting blood-brain barrier disruption following cisplatin treatment in a mouse model of chemotherapy-associated cognitive impairment. Geroscience (2025) 47(3):3835–47. 10.1007/s11357-025-01569-x 39982666 PMC12181602

[158] Flatters SJ Bennett GJ . Studies of peripheral sensory nerves in paclitaxel-induced painful peripheral neuropathy: evidence for mitochondrial dysfunction. Pain (2006) 122(3):245–57. 10.1016/j.pain.2006.01.037 16530964 PMC1805481

[159] Podratz JL Knight AM Ta LE Staff NP Gass JM Genelin K Cisplatin induced mitochondrial DNA damage in dorsal root ganglion neurons. Neurobiol Dis (2011) 41(3):661–8. 10.1016/j.nbd.2010.11.017 21145397 PMC3031677

[160] Tomaszewski A Busselberg D . Cisplatin modulates voltage gated channel currents of dorsal root ganglion neurons of rats. Neurotoxicology (2007) 28(1):49–58. 10.1016/j.neuro.2006.07.005 16945417

[161] Akin EJ Alsaloum M Higerd GP Liu S Zhao P Dib-Hajj FB Paclitaxel increases axonal localization and vesicular trafficking of Nav1.7. Brain (2021) 144(6):1727–37. 10.1093/brain/awab113 33734317 PMC8320304

[162] Pease-Raissi SE Pazyra-Murphy MF Li Y Wachter F Fukuda Y Fenstermacher SJ Paclitaxel reduces axonal bclw to initiate IP(3)R1-Dependent axon degeneration. Neuron (2017) 96(2):373–86 e6. 10.1016/j.neuron.2017.09.034 29024661 PMC5680044

[163] Yang IH Siddique R Hosmane S Thakor N Höke A . Compartmentalized microfluidic culture platform to study mechanism of paclitaxel-induced axonal degeneration. Exp Neurology (2009) 218(1):124–8. 10.1016/j.expneurol.2009.04.017 19409381 PMC4440669

[164] von Banchet GS Kiehl M Schaible HG . Acute and long-term effects of IL-6 on cultured dorsal root ganglion neurones from adult rat. J Neurochemistry (2005) 94(1):238–48. 10.1111/j.1471-4159.2005.03185.x 15953366

[165] Song DD Li Y Tang D Huang LY Yuan YZ . Neuron-glial communication mediated by TNF-Alpha and glial activation in dorsal root ganglia in visceral inflammatory hypersensitivity. Am J Physiol Gastrointest Liver Physiol (2014) 306(9):G788–95. 10.1152/ajpgi.00318.2013 24627565

[166] Deiteren A van der Linden L de Wit A Ceuleers H Buckinx R Timmermans JP P2X3 receptors mediate visceral hypersensitivity during acute chemically-induced colitis and in the post-inflammatory phase *via* different mechanisms of sensitization. PLoS One (2015) 10(4):e0123810. 10.1371/journal.pone.0123810 25885345 PMC4401691

[167] Valdez-Morales EE Sanchez-Navarro CA Reyes-Pavon D Barrios-Garcia T Ochoa-Cortes F Barajas-Espinosa A TNF-Alpha enhances sensory DRG neuron excitability through modulation of P2X3 receptors in an acute colitis model. Front Immunol (2022) 13:872760. 10.3389/fimmu.2022.872760 36032155 PMC9416886

[168] Lopez-Tofino Y Lopez-Gomez L Martin-Ruiz M Uranga JA Nurgali K Vera G Effects of repeated cisplatin and monosodium glutamate on visceral sensitivity in rats. Cells (2024) 14(1):26. 10.3390/cells14010026 39791727 PMC11719532

[169] Chen MX Chen Y Fu R Liu SY Yang QQ Shen TB . Activation of 5-HT and NR2B contributes to visceral hypersensitivity in irritable bowel syndrome in rats. Am J Transl Res (2016) 8(12):5580–90. 28078028 PMC5209508

[170] El-Ayache N Galligan JJ . 5-HT(3) receptor signaling in serotonin transporter-knockout rats: a female sex-specific animal model of visceral hypersensitivity. Am J Physiol Gastrointest Liver Physiol (2019) 316(1):G132–G43. 10.1152/ajpgi.00131.2018 30359082 PMC6383387

[171] Cirrincione AM Reimonn CA Harrison BJ Rieger S . Longitudinal RNA sequencing of skin and DRG neurons in mice with paclitaxel-induced peripheral neuropathy. Data (Basel) (2022) 7(6):72. 10.3390/data7060072 36248261 PMC9564132

[172] Cirrincione AM Pellegrini AD Dominy JR Benjamin ME Utkina-Sosunova I Lotti F Paclitaxel-induced peripheral neuropathy is caused by epidermal ROS and mitochondrial damage through conserved MMP-13 activation. Sci Rep (2020) 10(1):3970. 10.1038/s41598-020-60990-8 32132628 PMC7055229

[173] Huang TY Yang SS Liao CL Lin MH Lin HH Lin JC SPAK deficiency attenuates chemotherapy-induced intestinal mucositis. Front Oncol (2021) 11:733555. 10.3389/fonc.2021.733555 34888232 PMC8649624

[174] Tang D Qiu R Qiu X Sun M Su M Tao Z Dietary restriction rescues 5-fluorouracil-induced lethal intestinal toxicity in old mice by blocking translocation of opportunistic pathogens. Gut Microbes (2024) 16(1):2355693. 10.1080/19490976.2024.2355693 38780487 PMC11123560

[175] Ramakrishna C Corleto J Ruegger PM Logan GD Peacock BB Mendonca S Dominant role of the gut microbiota in chemotherapy induced neuropathic pain. Sci Rep (2019) 9(1):20324. 10.1038/s41598-019-56832-x 31889131 PMC6937259

[176] Zhong S Zhou Z Liang Y Cheng X Li Y Teng W Targeting strategies for chemotherapy-induced peripheral neuropathy: does gut microbiota play a role? Crit Rev Microbiol (2019) 45:1–25. 10.1080/1040841X.2019.1608905 31106639

[177] Gorgun MF Zhuo M Englander EW . Cisplatin toxicity in dorsal root ganglion neurons is relieved by meclizine *via* diminution of mitochondrial compromise and improved clearance of DNA damage. Mol Neurobiol (2017) 54(10):7883–95. 10.1007/s12035-016-0273-9 27858292 PMC5440214

[178] Shatunova S Aktar R Peiris M Lee JYP Vetter I Starobova H . The role of the gut microbiome in neuroinflammation and chemotherapy-induced peripheral neuropathy. Eur Journal Pharmacology (2024) 979:176818. 10.1016/j.ejphar.2024.176818 39029779

[179] Novick J Miner P Krause R Glebas K Bliesath H Ligozio G A randomized, double-blind, placebo-controlled trial of tegaserod in female patients suffering from irritable bowel syndrome with constipation. Aliment Pharmacol Ther (2002) 16(11):1877–88. 10.1046/j.1365-2036.2002.01372.x 12390096

[180] Chey WD Pare P Viegas A Ligozio G Shetzline MA . Tegaserod for female patients suffering from IBS with mixed bowel habits or constipation: a randomized controlled trial. Am J Gastroenterol (2008) 103(5):1217–25. 10.1111/j.1572-0241.2008.01808.x 18477346

[181] Cardoso LM Pansani TN Hebling J de Souza Costa CA Basso FG . Chemotherapy drugs and inflammatory cytokines enhance matrix metalloproteinases expression by oral mucosa cells. Arch Oral Biol (2021) 127:105159. 10.1016/j.archoralbio.2021.105159 34022544

[182] Brierley SM Hibberd TJ Spencer NJ . Spinal afferent innervation of the Colon and rectum. Front Cell Neurosci (2018) 12:467. 10.3389/fncel.2018.00467 30564102 PMC6288476

[183] Hayakawa Y Sakitani K Konishi M Asfaha S Niikura R Tomita H Nerve growth factor promotes gastric tumorigenesis through aberrant cholinergic signaling. Cancer Cell (2017) 31(1):21–34. 10.1016/j.ccell.2016.11.005 27989802 PMC5225031

[184] Rabben HL Andersen GT Olsen MK Overby A Ianevski A Kainov D Neural signaling modulates metabolism of gastric cancer. iScience (2021) 24(2):102091. 10.1016/j.isci.2021.102091 33598644 PMC7869004

[185] Zhao CM Hayakawa Y Kodama Y Muthupalani S Westphalen CB Andersen GT Denervation suppresses gastric tumorigenesis. Sci Transl Med (2014) 6(250):250ra115. 10.1126/scitranslmed.3009569 25143365 PMC4374618

[186] Wang SY Yang ZJ Zhang Z Zhang H . Aprepitant in the prevention of vomiting induced by moderately and highly emetogenic chemotherapy. Asian Pac J Cancer Prev (2014) 15(23):10045–51. 10.7314/apjcp.2014.15.23.10045 25556423

[187] Zidan J Haim N Beny A Stein M Gez E Kuten A . Octreotide in the treatment of severe chemotherapy-induced diarrhea. Ann Oncol (2001) 12(2):227–9. 10.1023/a:1008372228462 11300329

[188] Maroun JA Anthony LB Blais N Burkes R Dowden SD Dranitsaris G Prevention and management of chemotherapy-induced diarrhea in patients with colorectal cancer: a consensus statement by the Canadian working group on chemotherapy-induced diarrhea. Curr Oncol (2007) 14(1):13–20. 10.3747/co.2007.96 17576459 PMC1891194

[189] Lenfers BH Loeffler TM Droege CM Hausamen TU . Substantial activity of budesonide in patients with irinotecan (CPT-11) and 5-fluorouracil induced diarrhea and failure of loperamide treatment. Ann Oncol (1999) 10(10):1251–3. 10.1023/a:1008390308416 10586346

[190] Karthaus M Ballo H Abenhardt W Steinmetz T Geer T Schimke J Prospective, double-blind, placebo-controlled, multicenter, randomized phase III study with orally administered budesonide for prevention of irinotecan (CPT-11)-induced diarrhea in patients with advanced colorectal cancer. Oncology (2005) 68(4-6):326–32. 10.1159/000086971 16020959

[191] de Paula Eduardo F Bezinelli LM da Graca Lopes RM Nascimento Sobrinho JJ Hamerschlak N Correa L . Efficacy of cryotherapy associated with laser therapy for decreasing severity of melphalan-induced oral mucositis during hematological stem-cell transplantation: a prospective clinical study. Hematol Oncol (2015) 33(3):152–8. 10.1002/hon.2133 24519448

[192] Spielberger R Stiff P Bensinger W Gentile T Weisdorf D Kewalramani T Palifermin for oral mucositis after intensive therapy for hematologic cancers. N Engl J Med (2004) 351(25):2590–8. 10.1056/NEJMoa040125 15602019

[193] Ventafridda V Saita L Ripamonti C De Conno F . WHO guidelines for the use of analgesics in cancer pain. Int J Tissue React (1985) 7(1):93–6. 2409039

[194] Hardi H Estuworo GK Louisa M . Effectivity of oral ginger supplementation for chemotherapy induced nausea and vomiting (CINV) in children: a systematic review of clinical trials. J Ayurveda Integr Med (2024) 15(4):100957. 10.1016/j.jaim.2024.100957 39173346 PMC11388353

[195] Gupta K Walton R Kataria SP . Chemotherapy-induced nausea and vomiting: pathogenesis, recommendations, and new trends. Cancer Treat Res Commun (2021) 26:100278. 10.1016/j.ctarc.2020.100278 33360668

[196] Rojas C Raje M Tsukamoto T Slusher BS . Molecular mechanisms of 5-HT(3) and NK(1) receptor antagonists in prevention of emesis. Eur Journal Pharmacology (2014) 722:26–37. 10.1016/j.ejphar.2013.08.049 24184669

[197] Kaplan MA Prior MJ McKonly KI DuPont HL Temple AR Nelson EB . A multicenter randomized controlled trial of a liquid loperamide product *versus* placebo in the treatment of acute diarrhea in children. Clin Pediatr (Phila) (1999) 38(10):579–91. 10.1177/000992289903801003 10544864

[198] Thong JYH Satyavolu R . A case report of loperamide-induced respiratory depression in severe gastrointestinal inflammation secondary to chemotherapy. Cureus (2026) 18(2):e104460. 10.7759/cureus.104460 41924411 PMC13035859

